# Supplementary material for: Parvaxanthines D–F and Asponguanosines C and D, Racemic Natural Hybrids from the Insect Cyclopelta parva
Source: Molecules. 2021 Jun 9;26(12):3531. doi: 10.3390/molecules26123531 (PMC8229431; doi:10.3390/molecules26123531)
Supplement: Supplementary file 1 [file molecules-26-03531-s001.zip › molecules-1247919-supplementary.pdf]

Parvaxanthines D−F and asponguanosines C and D, racemic natural hybrids from the insect *Cyclopelta parva*

Heng Chen [1,2,†], Yong-Ming Yan [2,†], Dai-Wei Wang [2] and Yong-Xian Cheng [1,2,3,*]

[1] Guangdong Pharmaceutical University, Guangzhou 510006, PR China; ch13871071689@163.com (H.C.)

[2] School of Pharmaceutical Sciences, Shenzhen University Health Science Center, Shenzhen, 518060, PR China; yanym@szu.edu.cn (Y.-M.Y.); dweiwang@foxmail.com (D.-W.W.); yxcheng@szu.edu.cn (Y.-X.C.)

[3] Guangdong Key Laboratory for Functional Substances in Medicinal Edible Resources and Healthcare Products, School of Life sciences and Food Engineer, Hanshan Normal University, Chaozhou 521041, PR China

[†] These authors contributed equally to this work.

* Correspondence: yxcheng@szu.edu.cn (Y.-X.C.)

Content

Figure S1. ^{1}H NMR spectrum of **1** in DMSO-*d*4.
Figure S2. ^{13}C NMR and DEPT spectra of **1** in methanol-*d*4.
Figure S3. ^{1}H-^{1}H COSY spectrum of **1** in DMSO-*d*6.
Figure S4. HSQC spectrum of **1** in DMSO-*d*6.
Figure S5. HMBC spectrum of **1** in DMSO-*d*6
Figure S6. HRESIMS of **1**.
Figure S7. CD spectrum of (+)-**1** in methanol.
Figure S8. CD spectrum of (–)-**1** in methanol.
Figure S9. The chiral HPLC chromatogram of compound **1**.
Figure S10. ^{1}H NMR spectrum of **2** in DMSO-*d*6.
Figure S11. ^{13}C NMR and DEPT spectra of **2** in DMSO-*d*6.
Figure S12. ^{1}H-^{1}H COSY spectrum of **2** in DMSO-*d*6.
Figure S13. HSQC spectrum of **2** in DMSO-*d*6.
Figure S14. HMBC spectrum of **2** in DMSO-*d*6.
Figure S15. ROESY spectrum of **2** in DMSO-*d*6.
Figure S16. HRESIMS of **2**.
Figure S17. CD spectrum of (+)-**2** in methanol.
Figure S18. CD spectrum of (–)-**2** in methanol.
Figure S19. The chiral HPLC chromatogram of of compound **2**.
Figure S20. ^{1}H NMR spectrum of **3** in methanol-*d*6.
Figure S21. ^{13}C NMR and DEPT spectra of **3** in methanol-*d*6.
Figure S22. ^{1}H-^{1}H COSY spectrum of **3** in methanol-*d*6.
Figure S23. HSQC spectrum of **3** in methanol-*d*6.
Figure S24. HMBC spectrum of **3** in methanol-*d*6.
Figure S25. HRESIMS of **3**.
Figure S26. CD spectrum of (+)-**3** in methanol.
Figure S27. CD spectrum of (–)-**3** in methanol.

Figure S28. The chiral HPLC chromatogram of compound **3**.
Figure S29. ^{1}H NMR spectrum of **6** in methanol-d_4.
Figure S30. ^{13}C NMR and DEPT spectra of **6** in methanol-d_4.
Figure S31. ^{1}H-^{1}H COSY spectrum of **6** in methanol-d_4.
Figure S32. HSQC spectrum of **6** in methanol-d_4.
Figure S33. HMBC spectrum of **6** in methanol-d_4.
Figure S34. HRESIMS of **6**.
Figure S35. CD spectrum of **6** in methanol.
Figure S36. ^{1}H NMR spectrum of **7** in methanol-d_4.
Figure S37. ^{13}C NMR and DEPT spectra of **7** in methanol-d_4.
Figure S38. ^{1}H-^{1}H COSY spectrum of **7** in methanol-d_4.
Figure S39. HSQC spectrum of **7** in methanol-d_4.
Figure S40. HMBC spectrum of **7** in methanol-d_4.
Figure S41. HRESIMS of **7**.
Figure S42. CD spectrum of **7** in methanol.
Figure S43. HPLC analysis of the derivatives of compounds **6**, **7**, *D*-ribose, and *L*-ribose.
Figure S44. The chiral HPLC analysis of **6** by Daicel Chiralpak AD-H column.
Figure S45. The chiral HPLC analysis of **6** by Daicel Chiralpak IC column.
Figure S46. The chiral HPLC analysis of **6** by Daicel Chiralpak OD-H column.
Figure S47. The chairl HPLC analysis of **6** by Daicel Chiralpak Phenomenex column.
Figure S48. The chiral HPLC analysis of **7** by Daicel Chiralpak OD-H column.
Figure S49. The chiral HPLC analysis of **7** by Daicel Chiralpak IC column.
Figure S50. The chiral HPLC analysis of **7** by Daicel Chiralpak AD-H column.
Figure S51. The chairl HPLC analysis of **7** by Daicel Chiralpak Phenomenex column.
Firure S52. Anti-HSV-1/F activity assay results of compounds **1**−**7**.
Figure S53. Cytotoxicity of compounds **1**−**7** in naive T Cells.
Figure S54. Effect of compounds **1**−**7** on IFN- γ signaling in mouse T cells in vitro.
Figure S55. Effect of compounds **1**−**7** on T-cell proliferation and activation activated by Con A.
Figure S56. Cytotoxic effects of compounds **1**−**7** on human cancer cells.

1. Spectra of the new compounds **1**–**3** and **6**–**7**

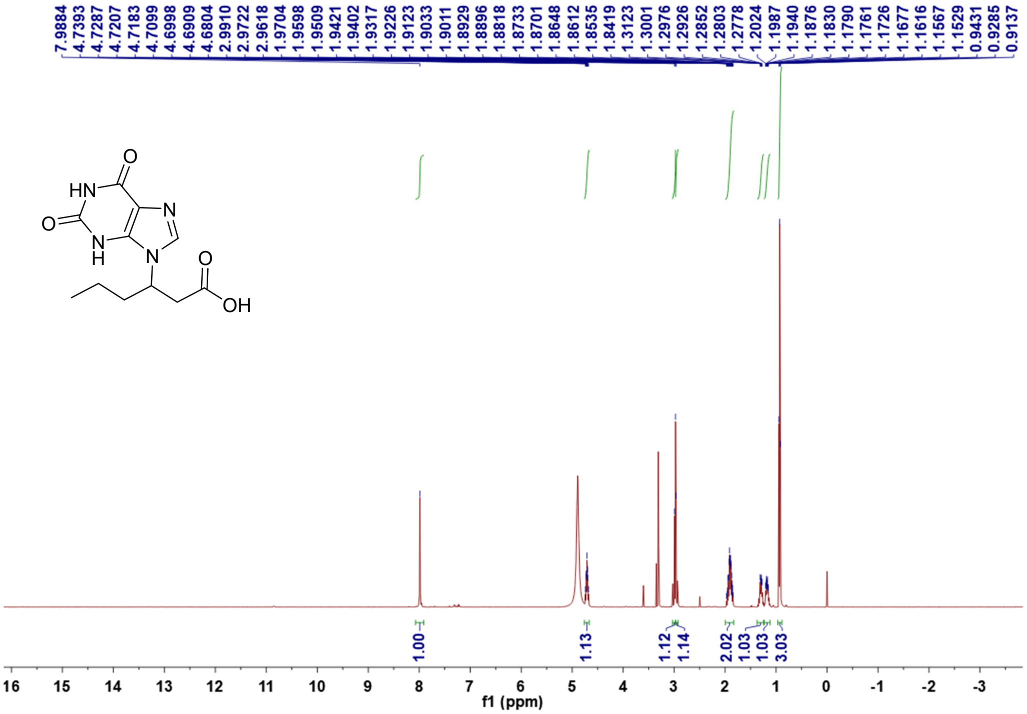

Figure S1. ^{1}H NMR spectrum of **1** in DMSO-*d*4.

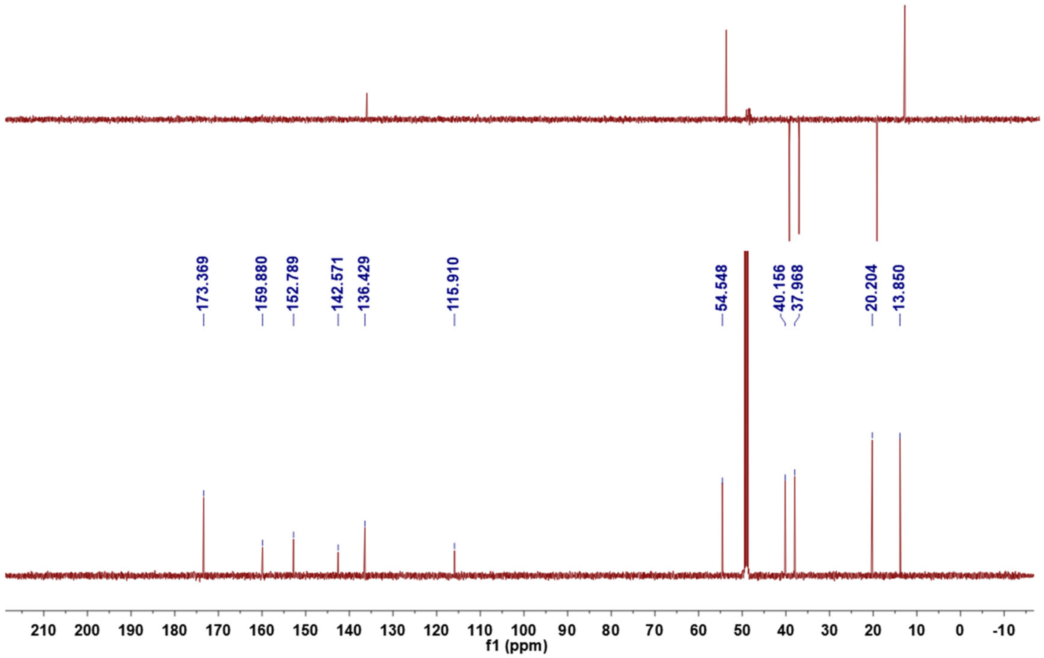

Figure S2. ^{13}C NMR and DEPT spectra of **1** in methanol-*d*4.

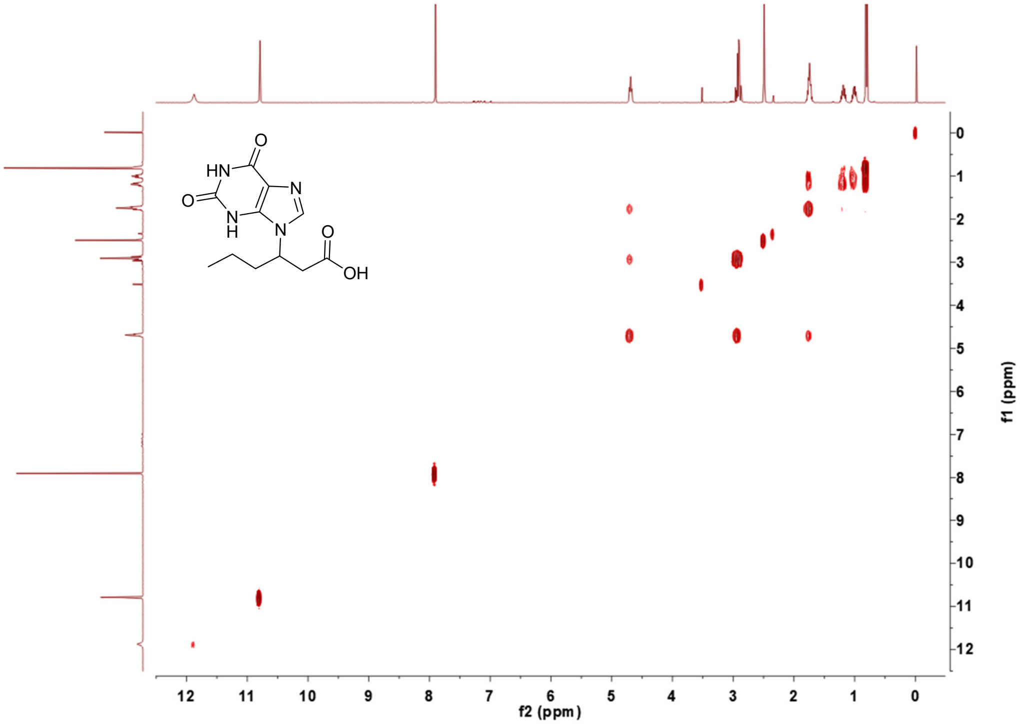

Figure S3. ^{1}H-^{1}H COSY spectrum of **1** in DMSO-d_6.

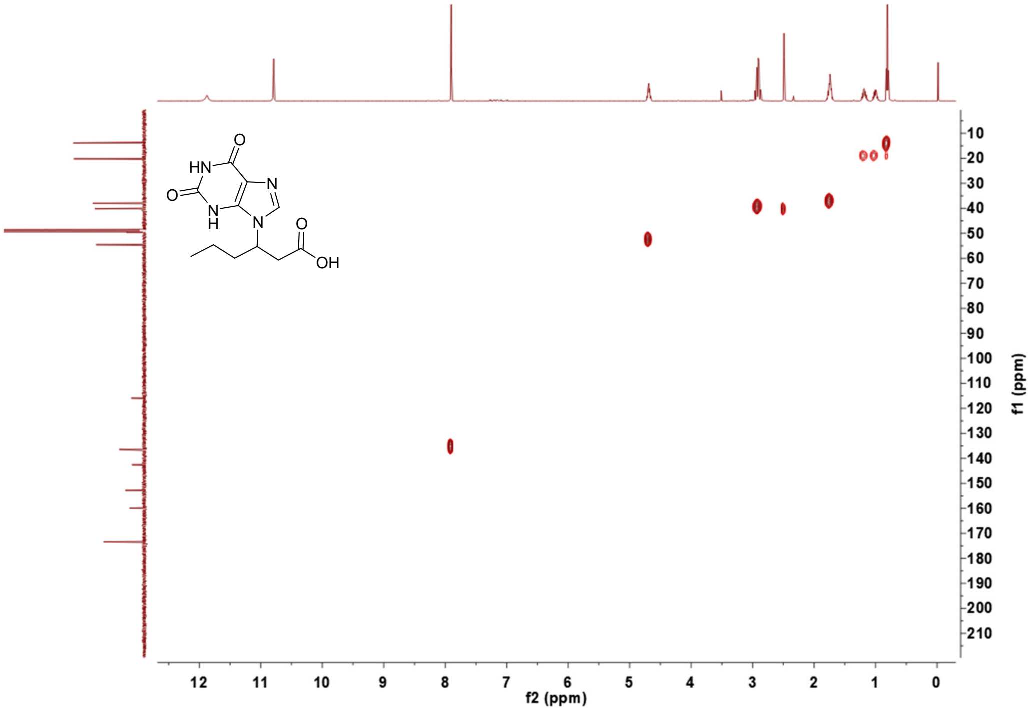

Figure S4. HSQC spectrum of **1** in DMSO-d_6.

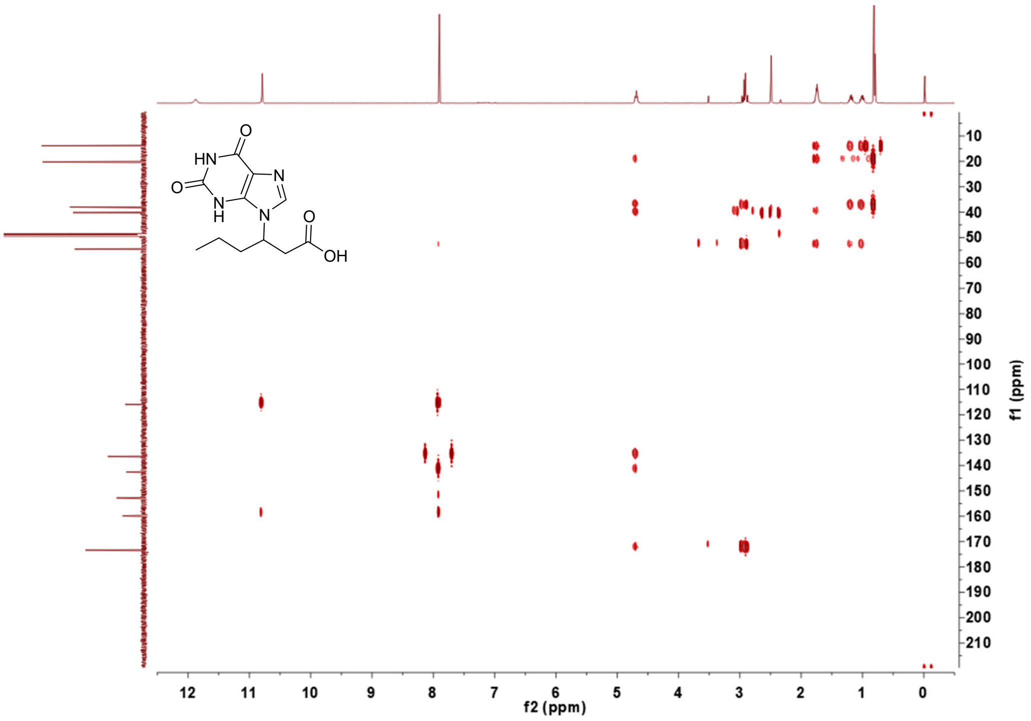

Figure S5. HMBC spectrum of **1** in DMSO-*d*$_6$.

Acquisition Date	28/8/2020 12:12:58 PM	Result Table	XXC-125
Acquisition Method	N/A	Algorithm Used	AutoPeak
Project	N/A	Instrument Name	X500 QTOF

Mass Spretra

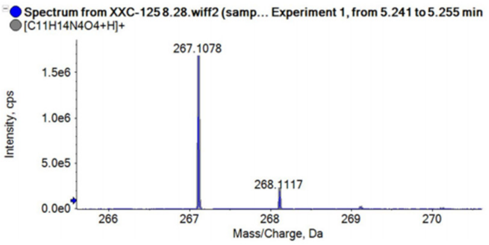

#	Analyte Peak Name	Formula	Precursor Mass	Found At Mass	Mass Error (ppm)
1	XXC-125	C11H14N4O4	267.1090	267.1078	-3.8

Figure S6. HRESIMS of **1**.

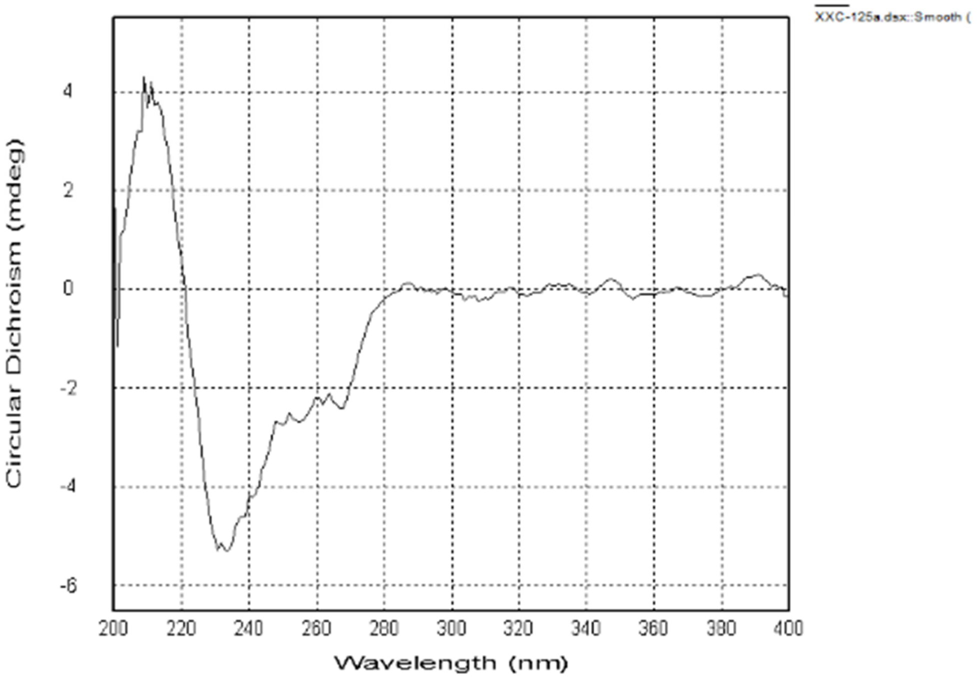

Figure S7. CD spectrum of (+)-**1** in methanol.

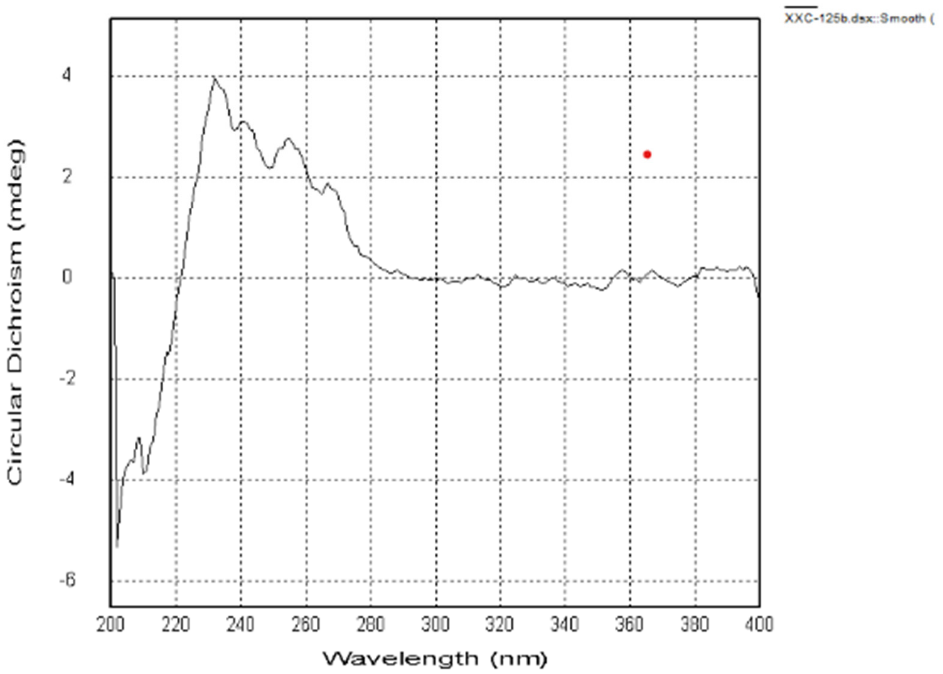

Figure S8. CD spectrum of (−)-**1** in methanol.

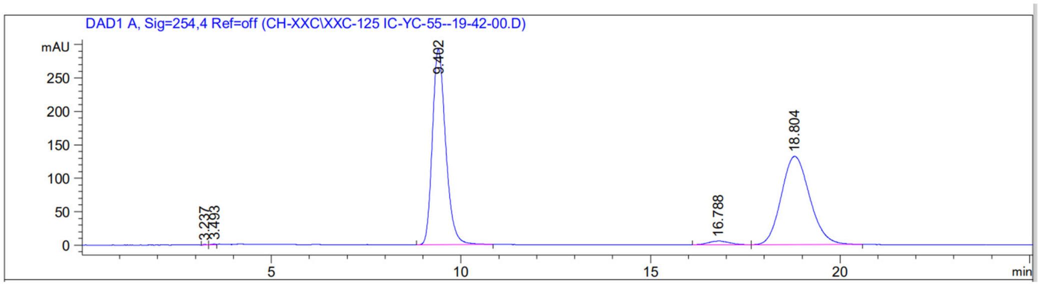

Figure S9. The HPLC chromatogram on chiral resolution of compound **1**.

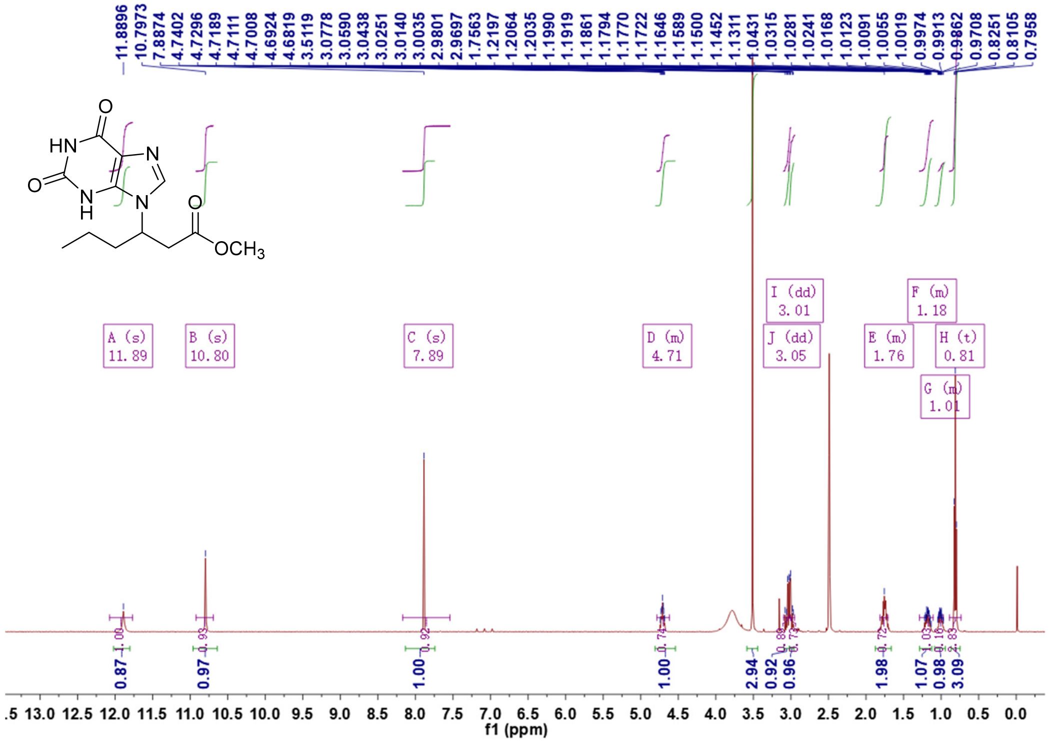

Figure S10. ^{1}H NMR spectrum of **2** in DMSO-*d*6.

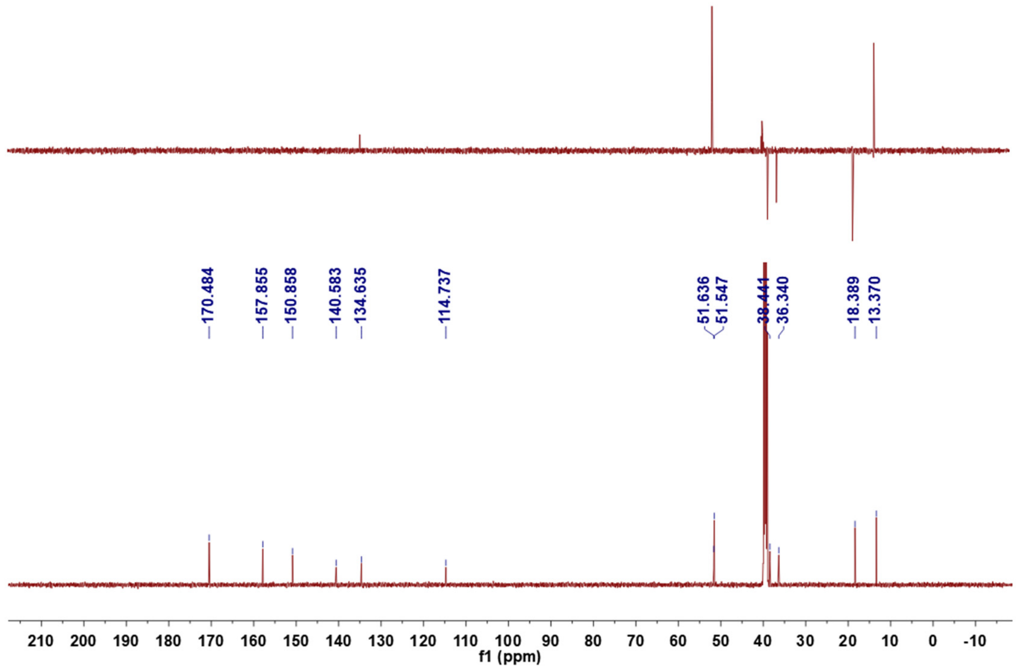

Figure S11. ^{13}C NMR and DEPT spectra of **2** in DMSO-*d*6.

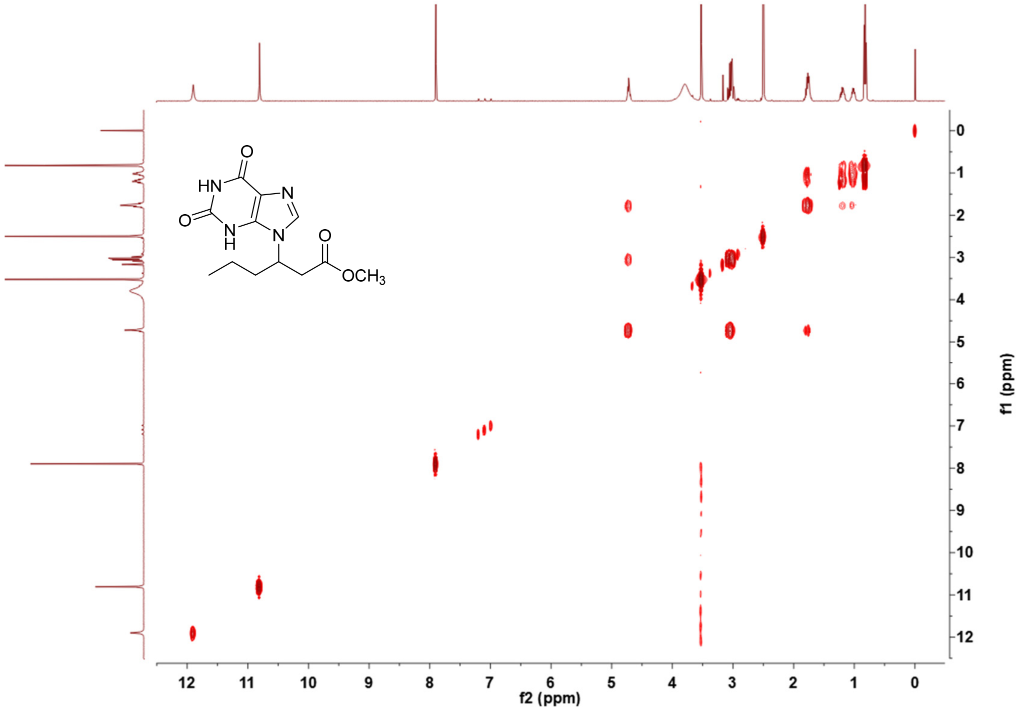

Figure S12. ^{1}H-^{1}H COSY spectrum of **2** in DMSO-*d*6.

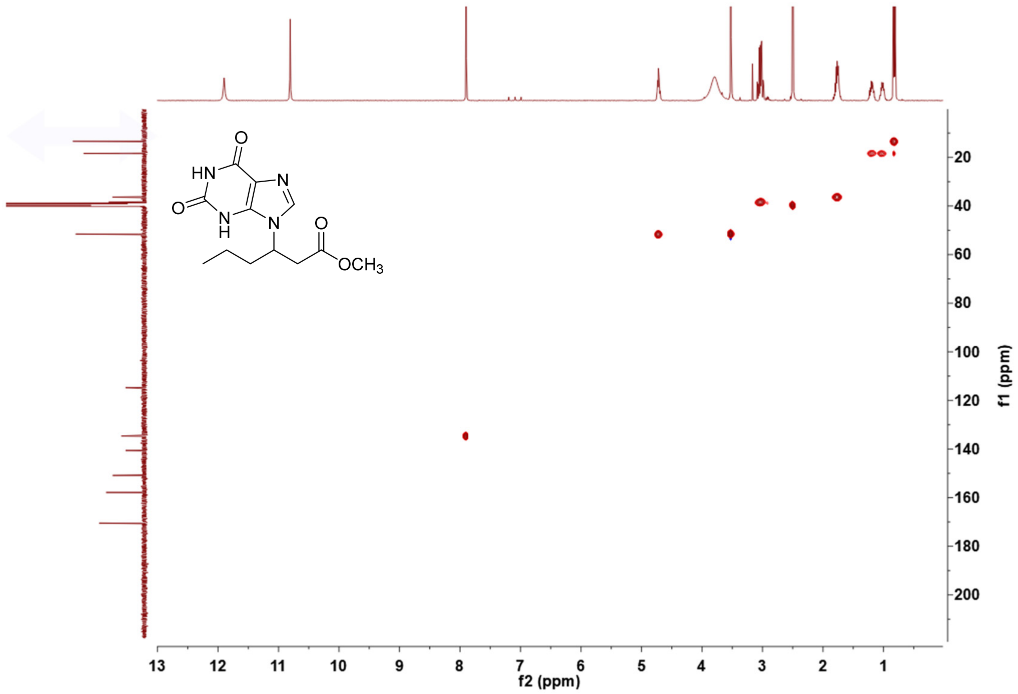

Figure S13**.** HSQC spectrum of **2** in DMSO-*d*6.

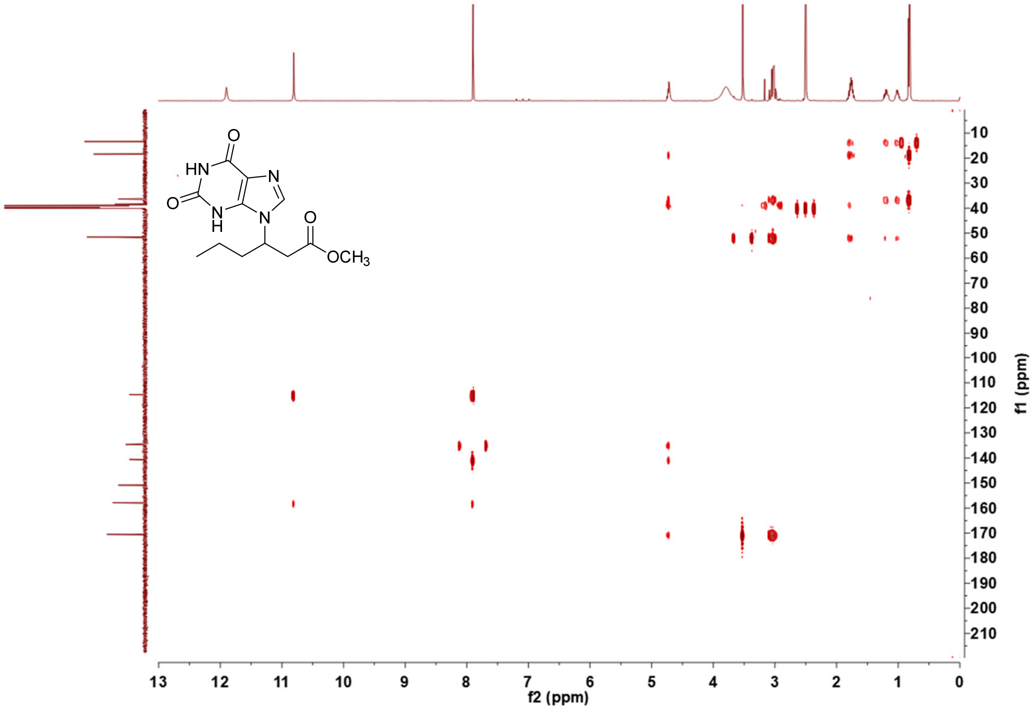

Figure S14. HMBC spectrum of **2** in DMSO-d_6.

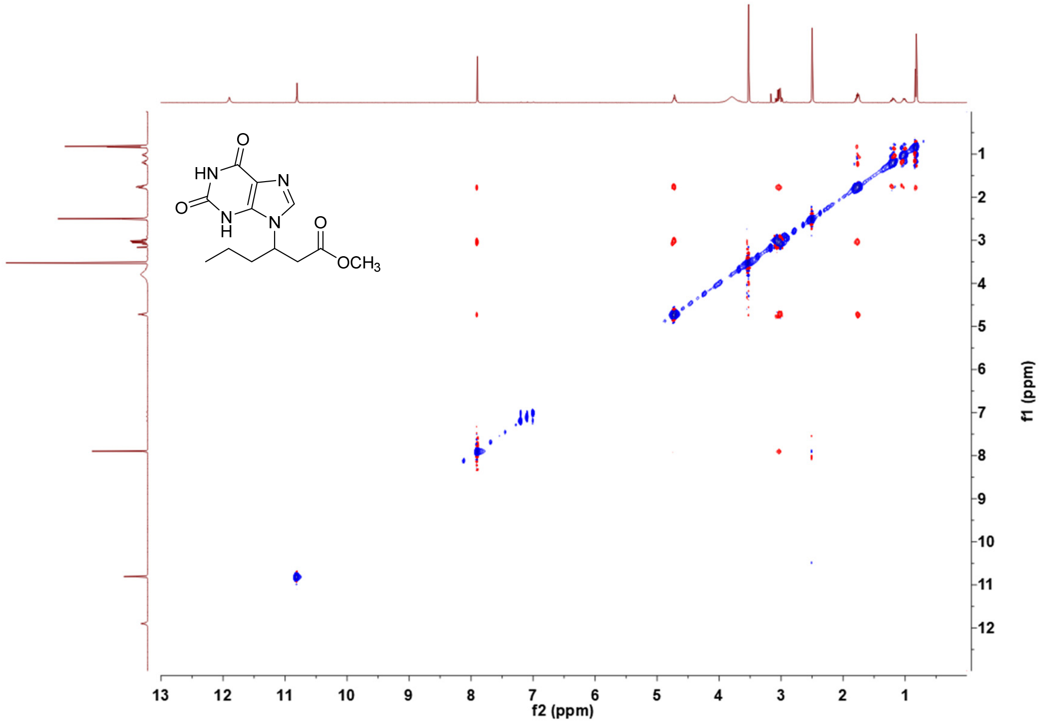

Figure S15. ROESY spectrum of **2** in DMSO-d_6.

Acquisition Date	28/8/2020 12:23:31 PM	Result Table	XXC-129
Acquisition Method	N/A	Algorithm Used	AutoPeak
Project	N/A	Instrument Name	X500 QTOF

Mass Spretra

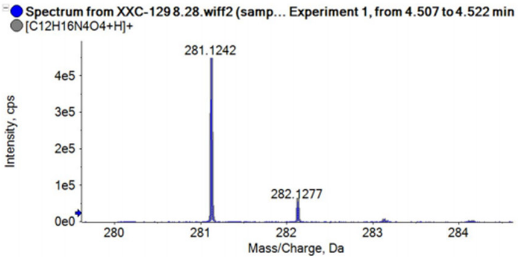

#	Analyte Peak Name	Formula	Precursor Mass	Found At Mass	Mass Error (ppm)
1	**XXC-129**	C12H16N4O4	281.1240	281.1242	-0.9

Figure S16. HRESIMS of **2.**

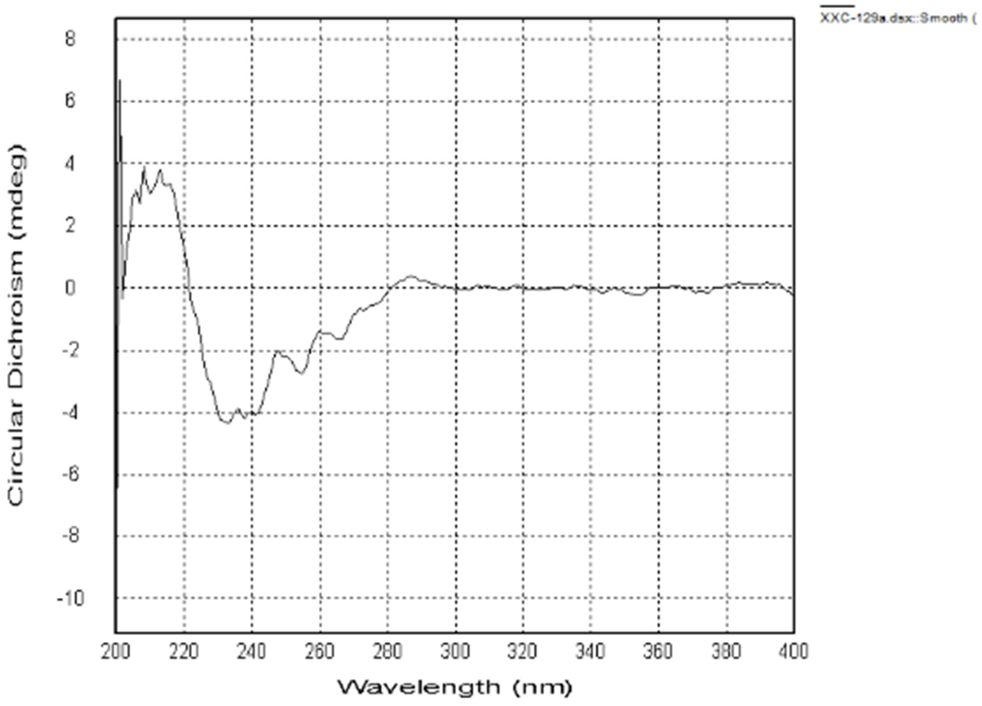

Figure S17. CD spectrum of (+)-**2** in methanol.

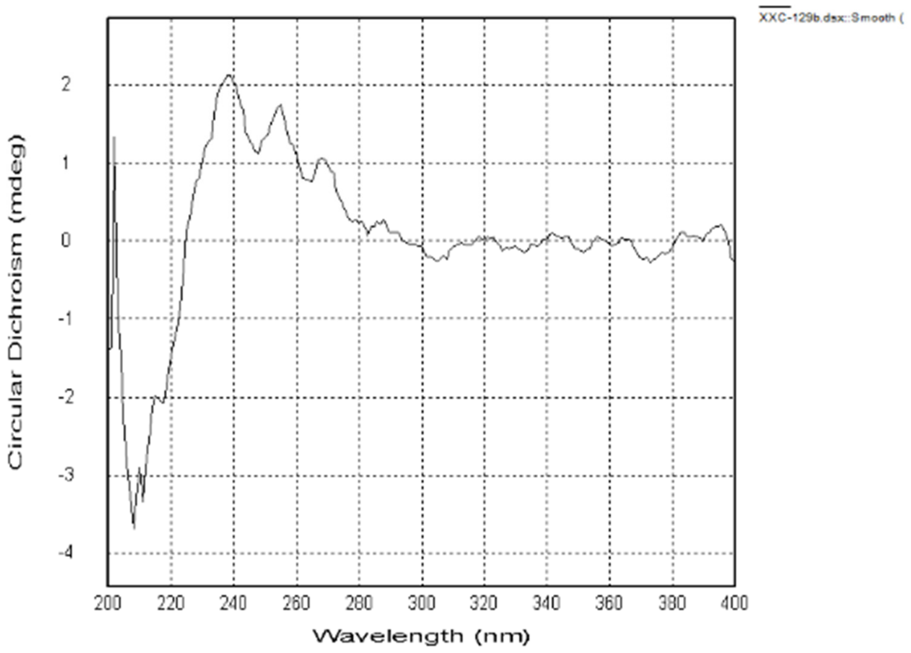

Figure S18. CD spectrum of (−)-**2** in methanol.

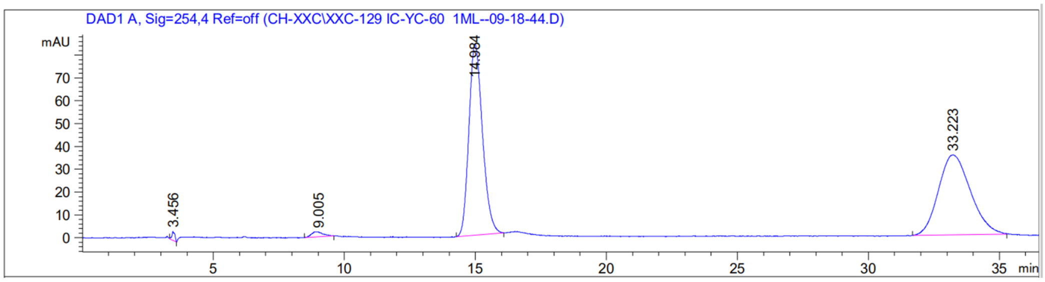

Figure S19. The HPLC chromatogram on chiral resolution of compound **2**.

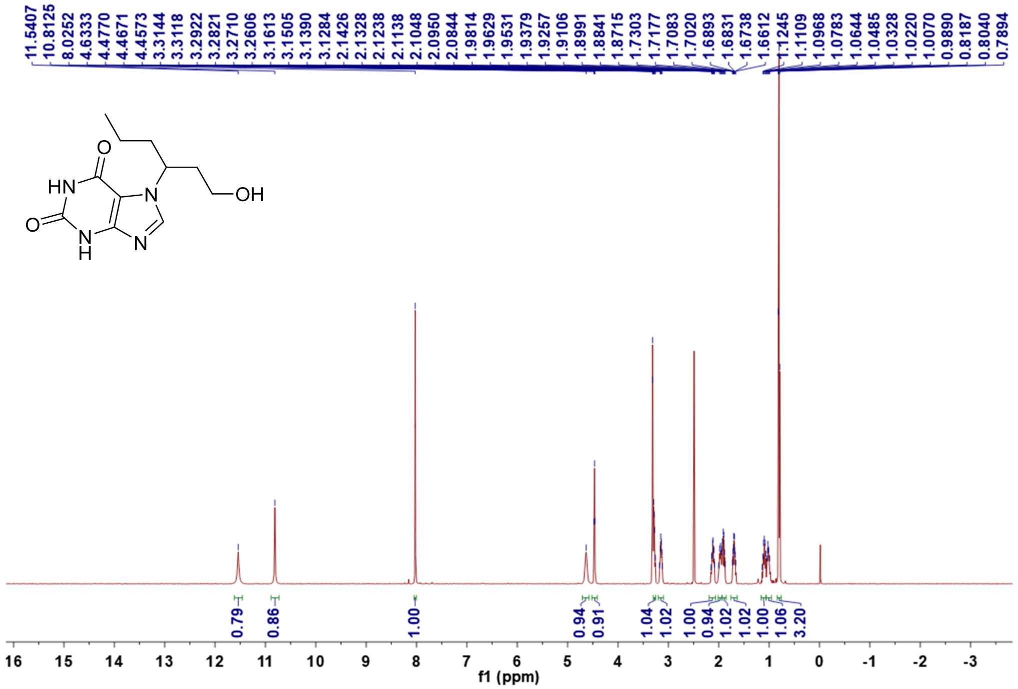

Figure S20. ^{1}H NMR spectrum of **3** in methanol-d_6.

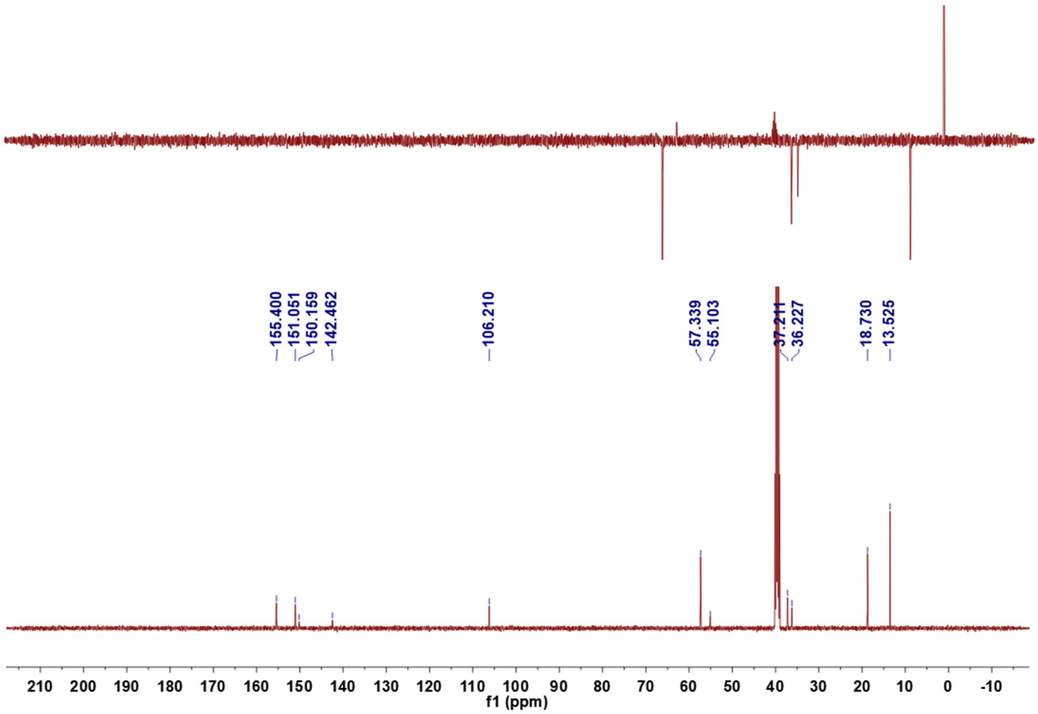

Figure S21. ^{13}C NMR and DEPT spectra of **3** in DMSO-d_6.

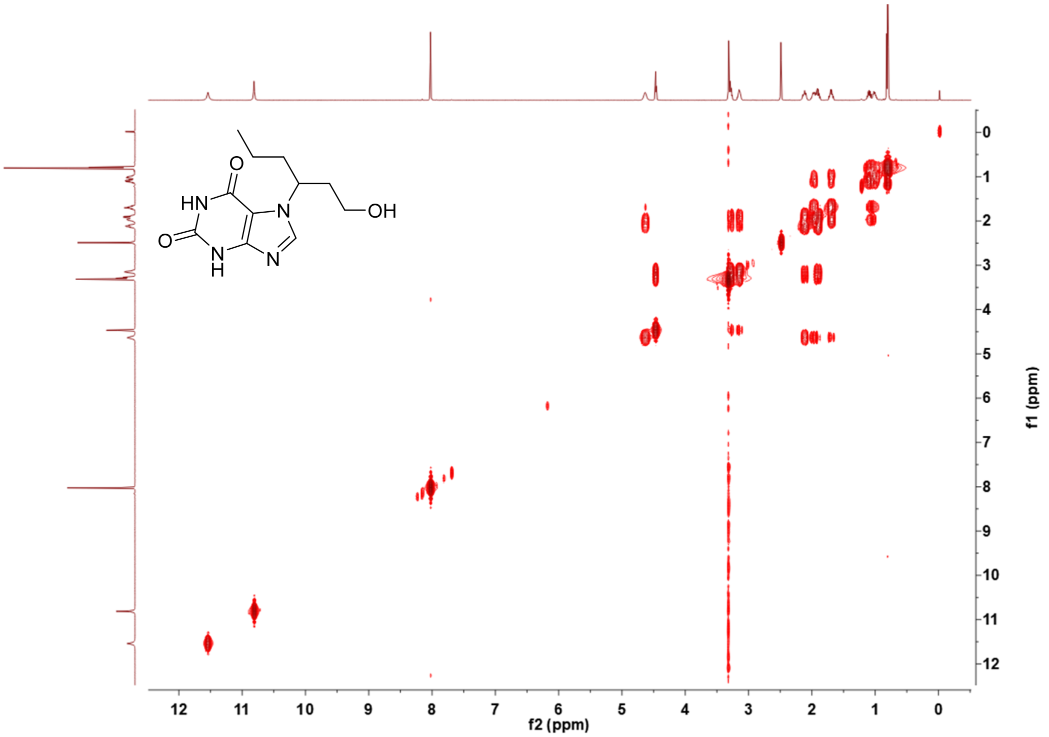

Figure S22. ^{1}H-^{1}H COSY spectrum of **3** in DMSO-d_6.

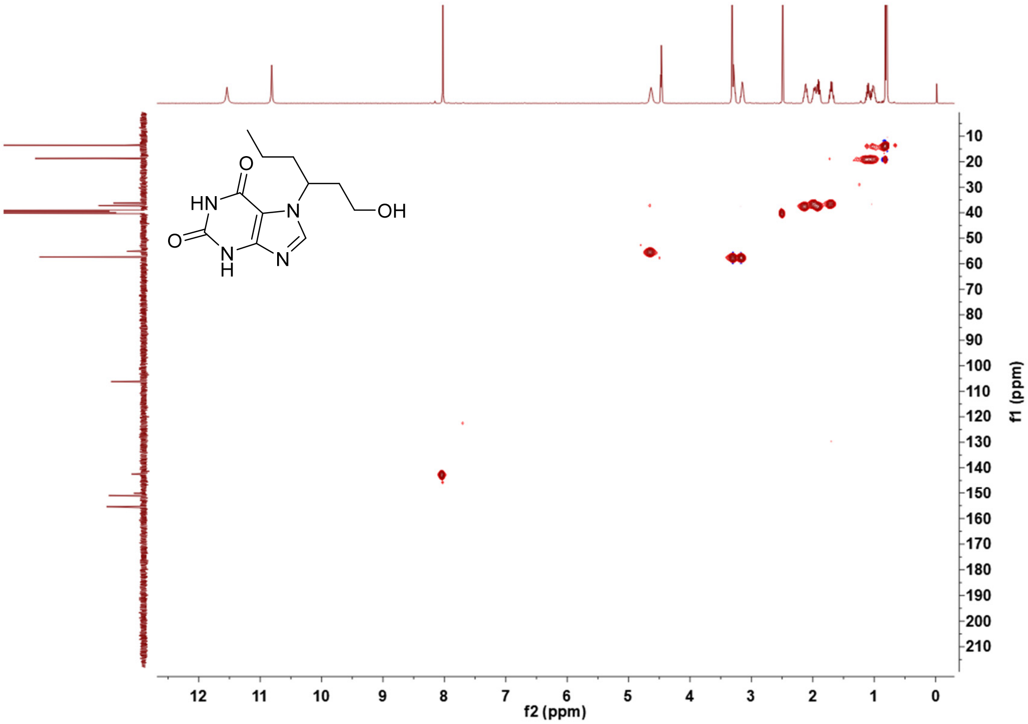

Figure S23. HSQC spectrum of **3** in DMSO-d_6.

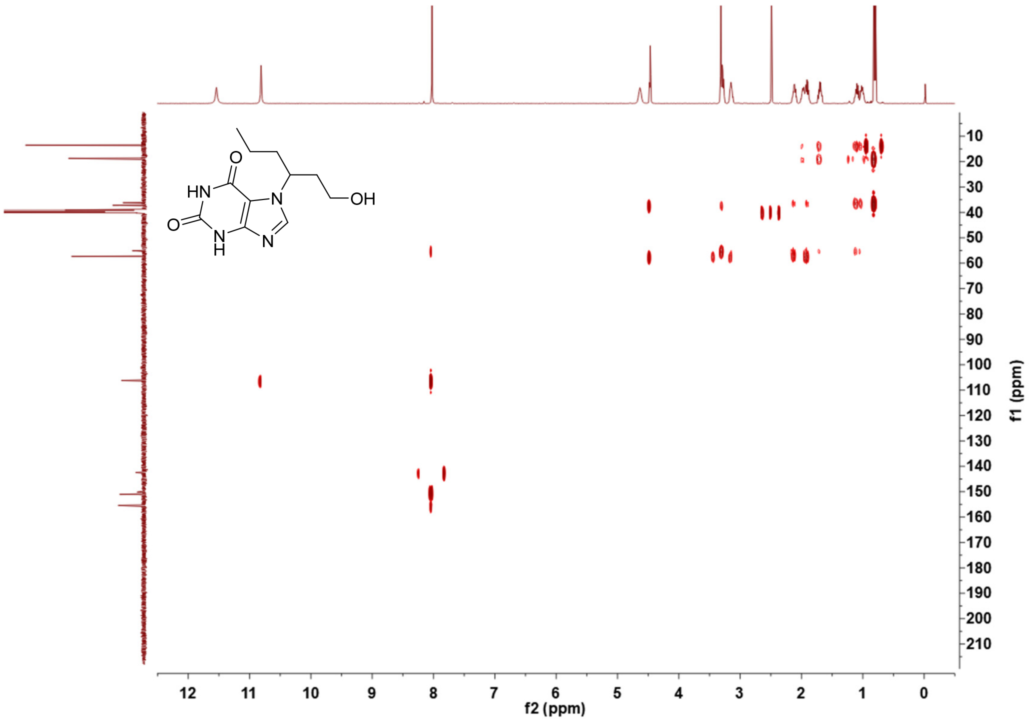

Figure S24. HMBC spectrum of **3** in DMSO-d_6.

**Acquisition Date**	28/8/2020 12:55:04 PM	**Result Table**	XXC-140
**Acquisition Method**	N/A	**Algorithm Used**	AutoPeak
**Project**	N/A	**Instrument Name**	X500 QTOF

Mass Spretra

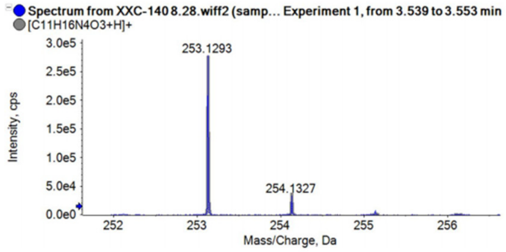

#	Analyte Peak Name	Formula	Precursor Mass	Found At Mass	Mass Error (ppm)
1	**XXC-140**	C11H16N4O3	253.1300	253.1293	-0.9

Figure S25. HRESIMS of **3**.

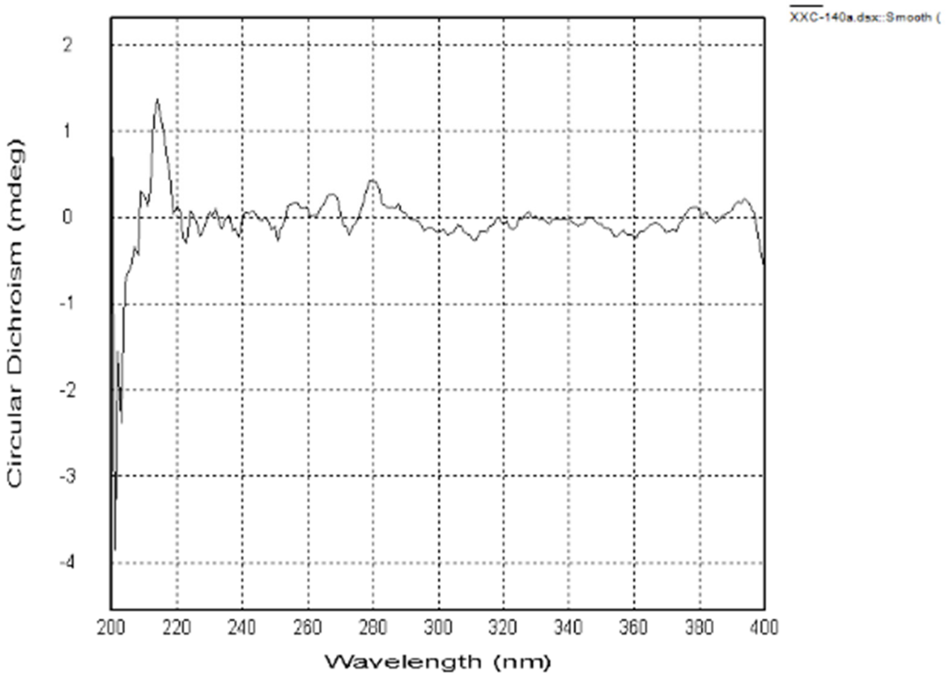

Figure S26. CD spectrum of (+)-**3** in methanol.

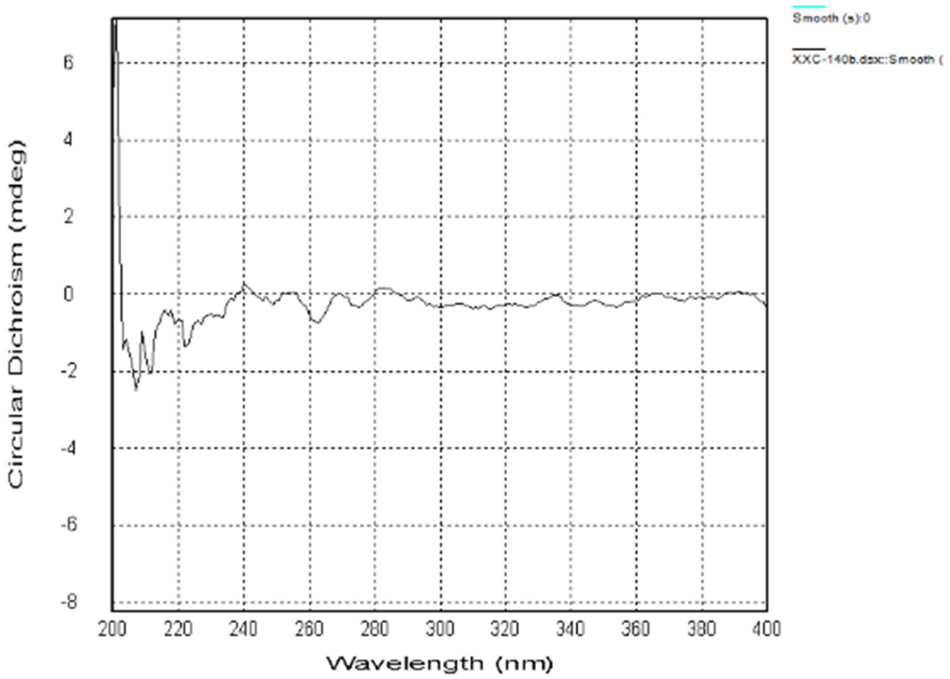

Figure S27. CD spectrum of (–)-**3** in methanol.

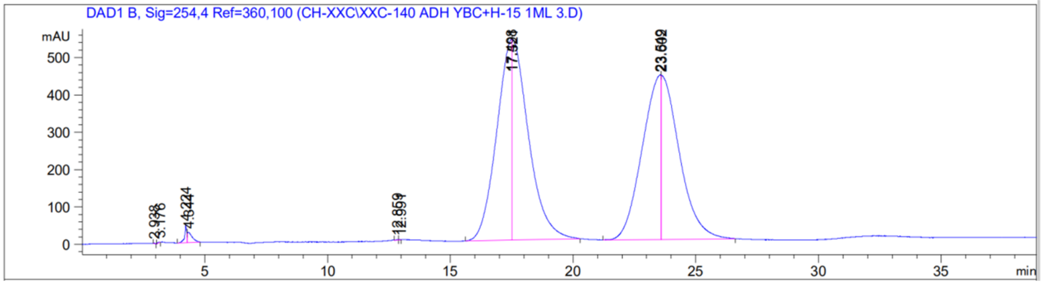

Figure S28. The HPLC chromatogram on chiral resolution of compound **3**.

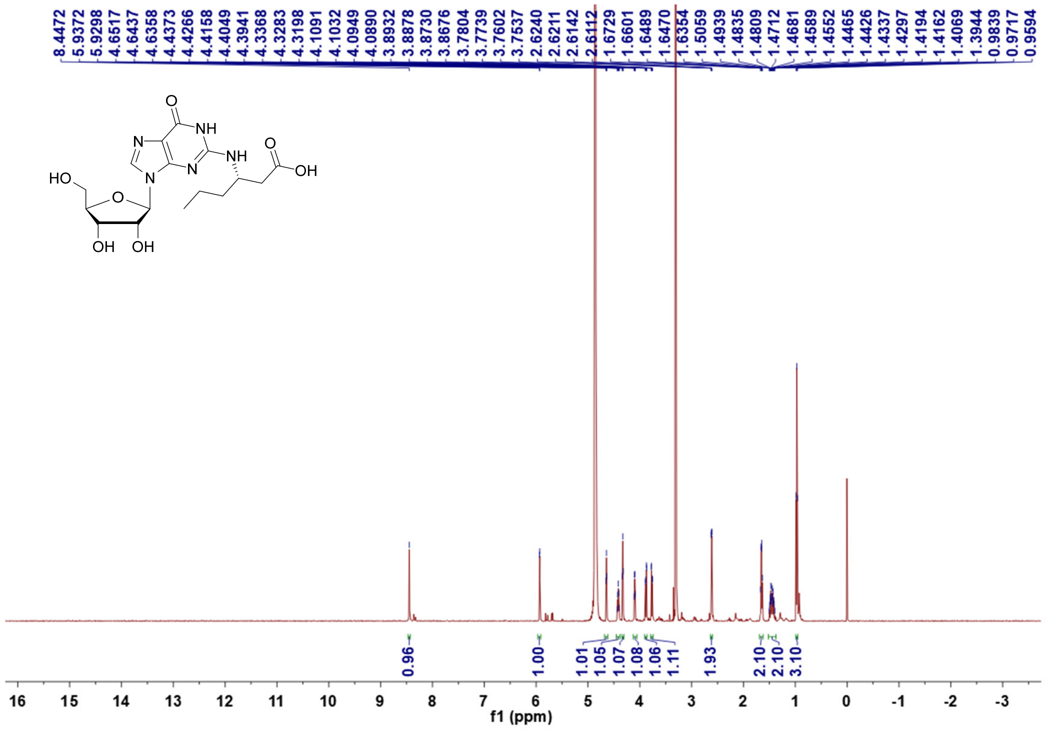

Figure S29. ^{1}H NMR spectrum of **6** in methanol-d_4.

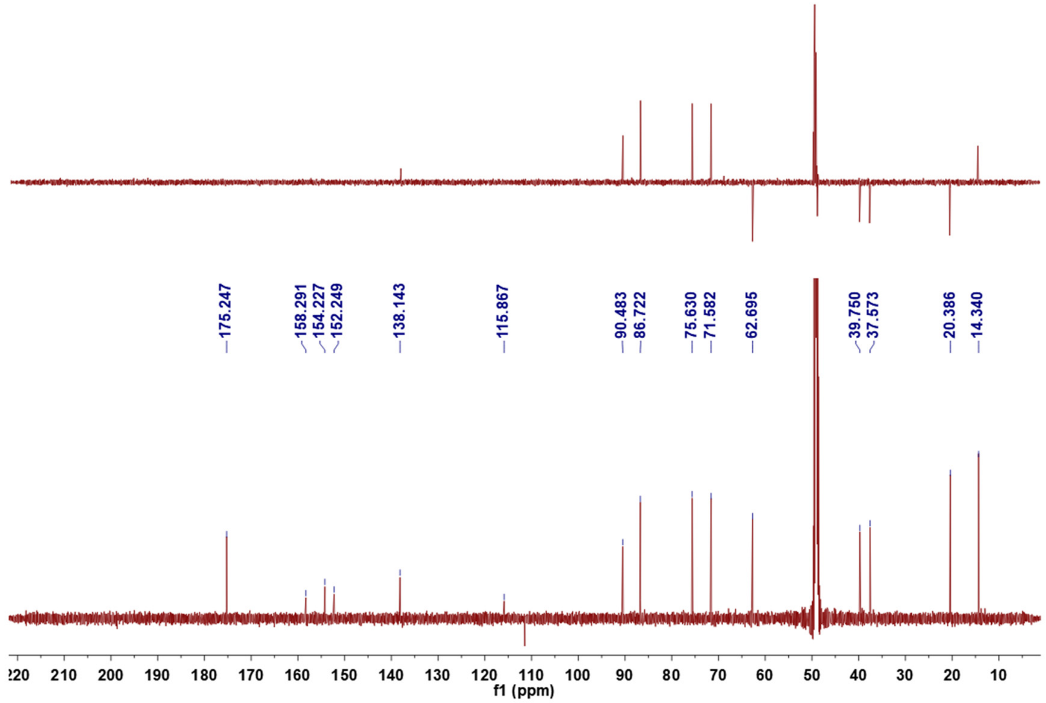

Figure S30. ^{13}C NMR and DEPT spectra of **6** in dmso-d_6.

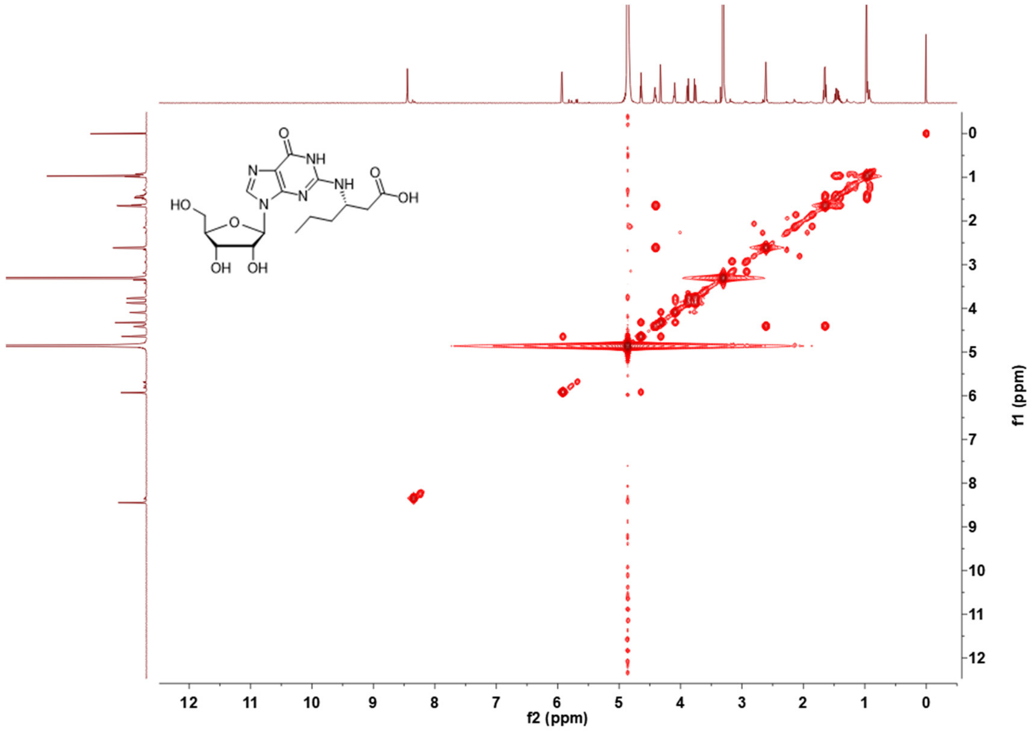

Figure S31. ^{1}H-^{1}H COSY spectrum of **6** in methanol-*d*4.

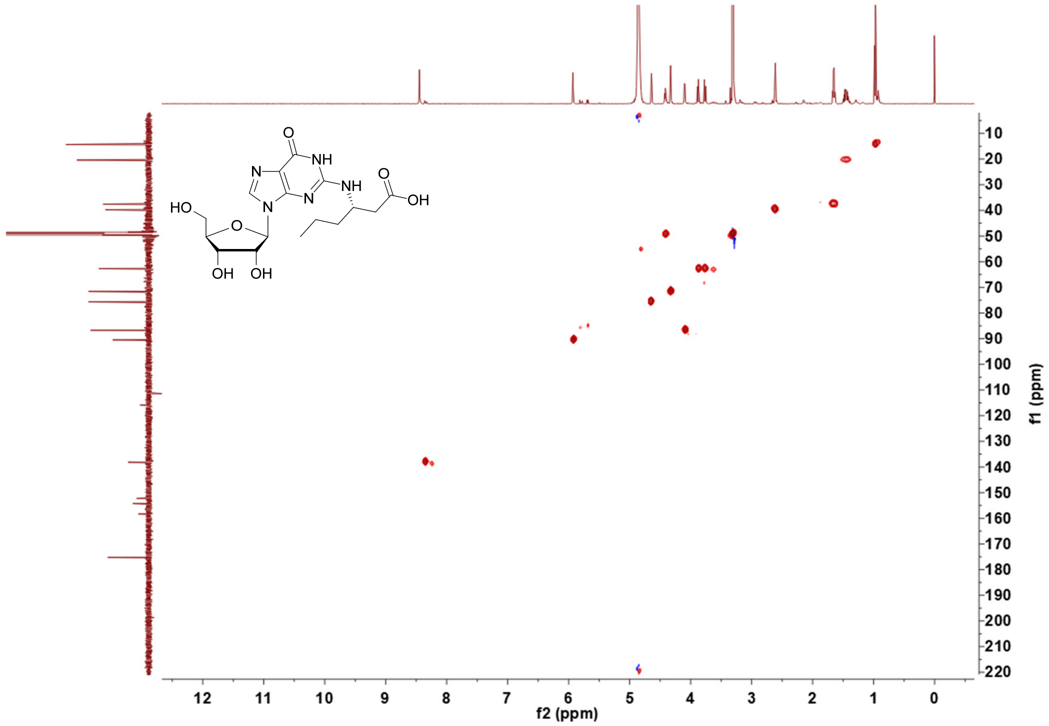

Figure S32. HSQC spectrum of **6** in methanol-*d*4.

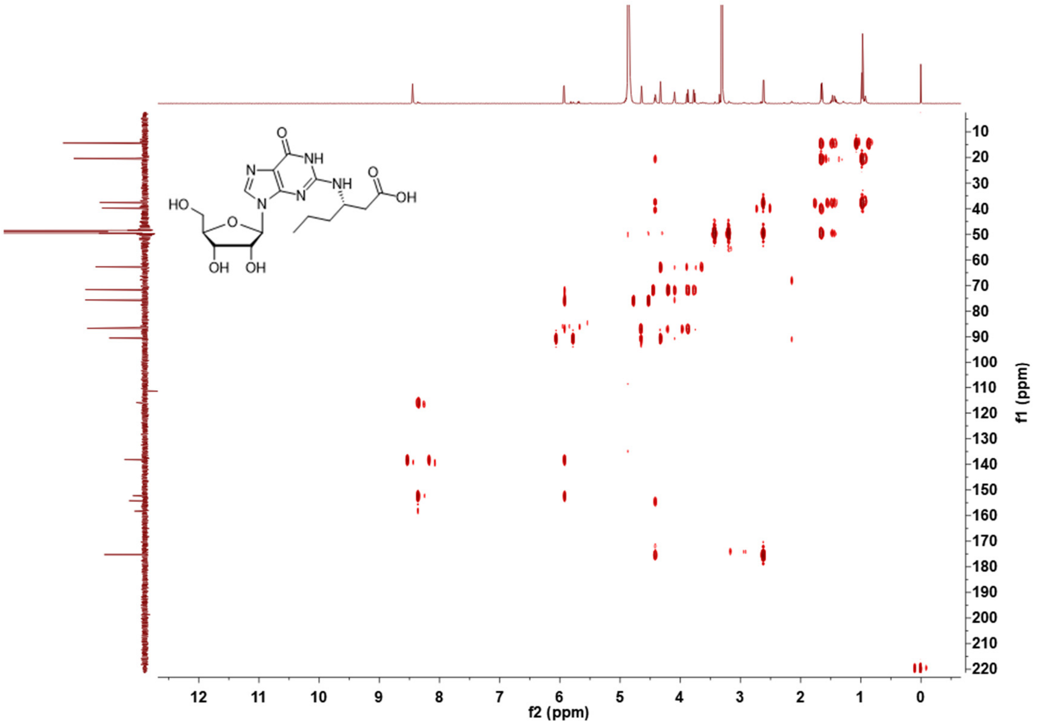

Figure S33. HMBC spectrum of **6** in methanol-*d*4.

Acquisition Date	28/8/2020 4:19:42 PM	Result Table	XXC-144
Acquisition Method	N/A	Algorithm Used	AutoPeak
Project	N/A	Instrument Name	X500 QTOF

Mass Spretra

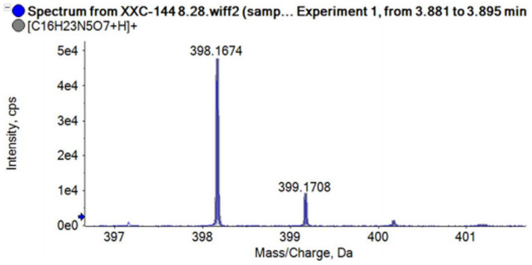

#	Analyte Peak Name	Formula	Precursor Mass	Found At Mass	Mass Error (ppm)
1	**XXC-144**	C16H23N5O7	398.1670	398.1674	1.0

Figure S34. HRESIMS of **6**.

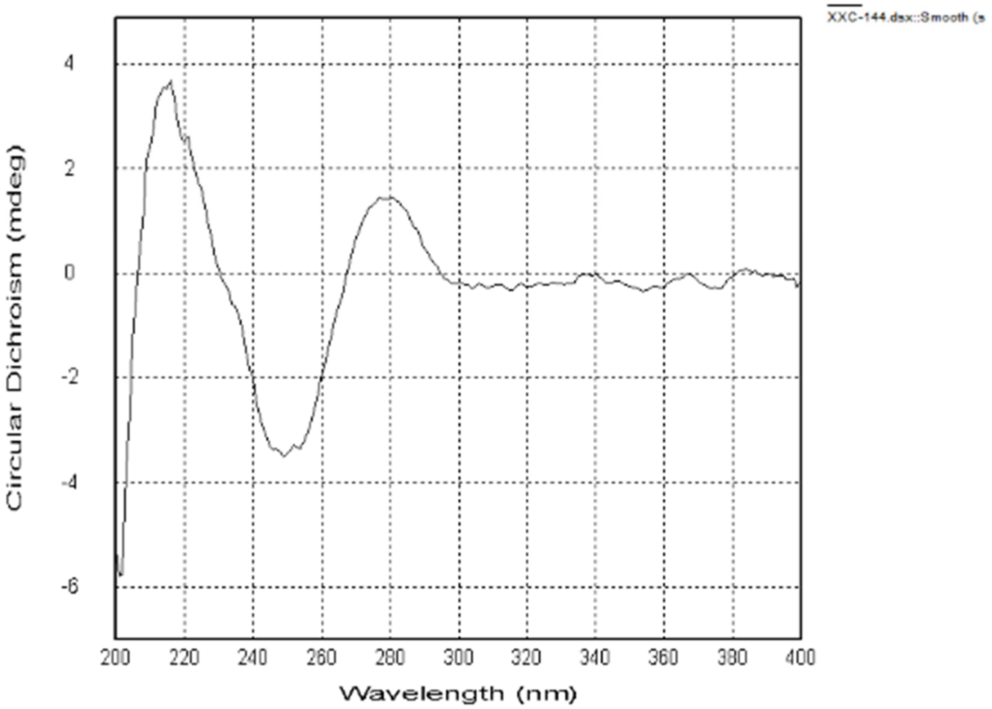

Figure S35. CD spectrum of **6** in methanol.

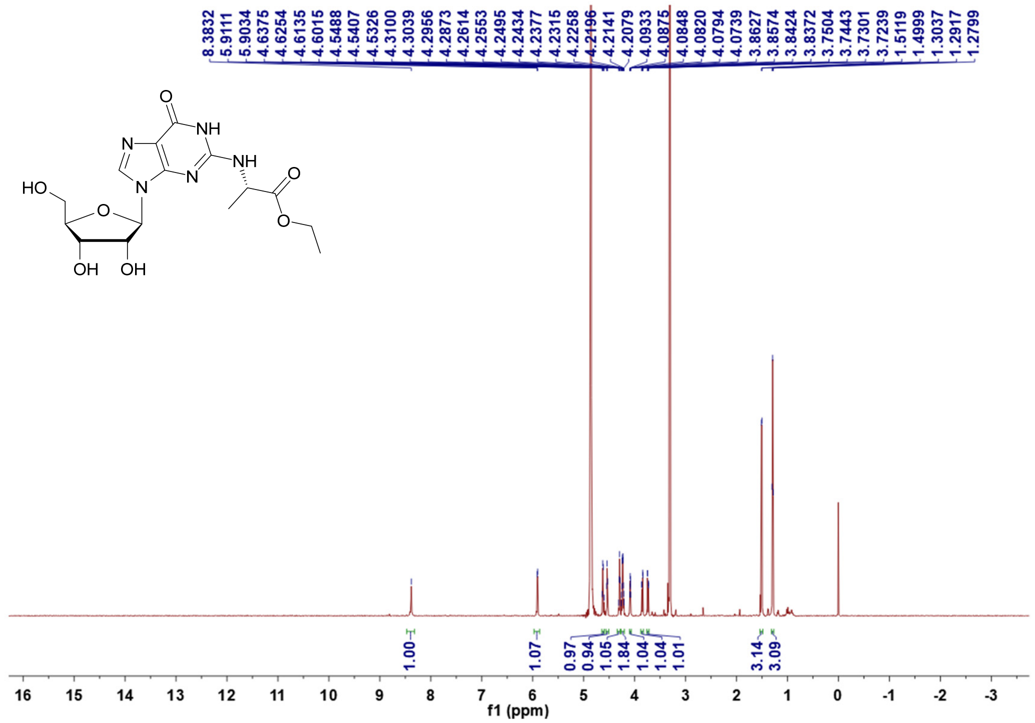

Figure S36. ^{1}H NMR spectrum of **7** in methanol-d_4.

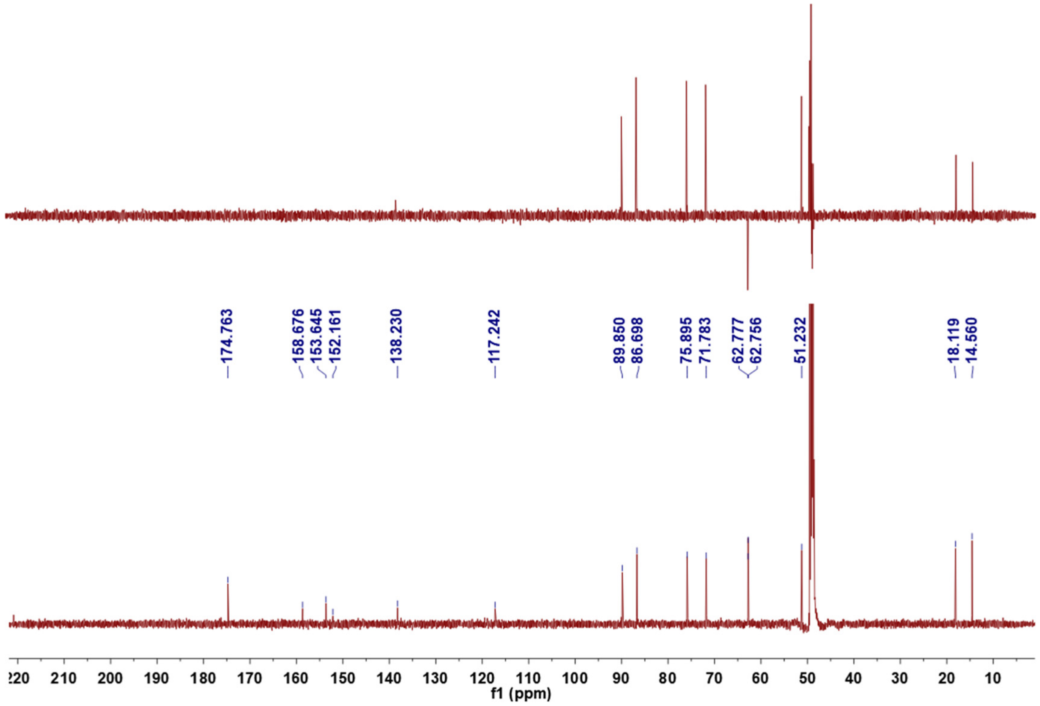

Figure S37. ^{13}C NMR and DEPT spectra of **7** in methanol-d_4.

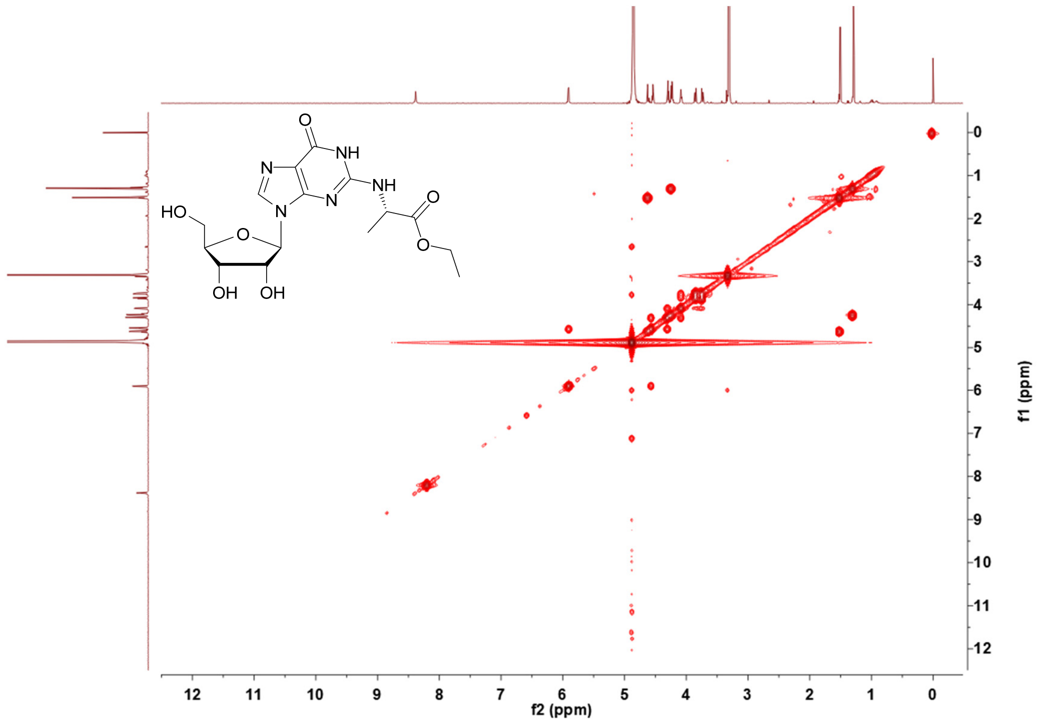

Figure S38. ^{1}H-^{1}H COSY spectrum of **7** in methanol-d_4.

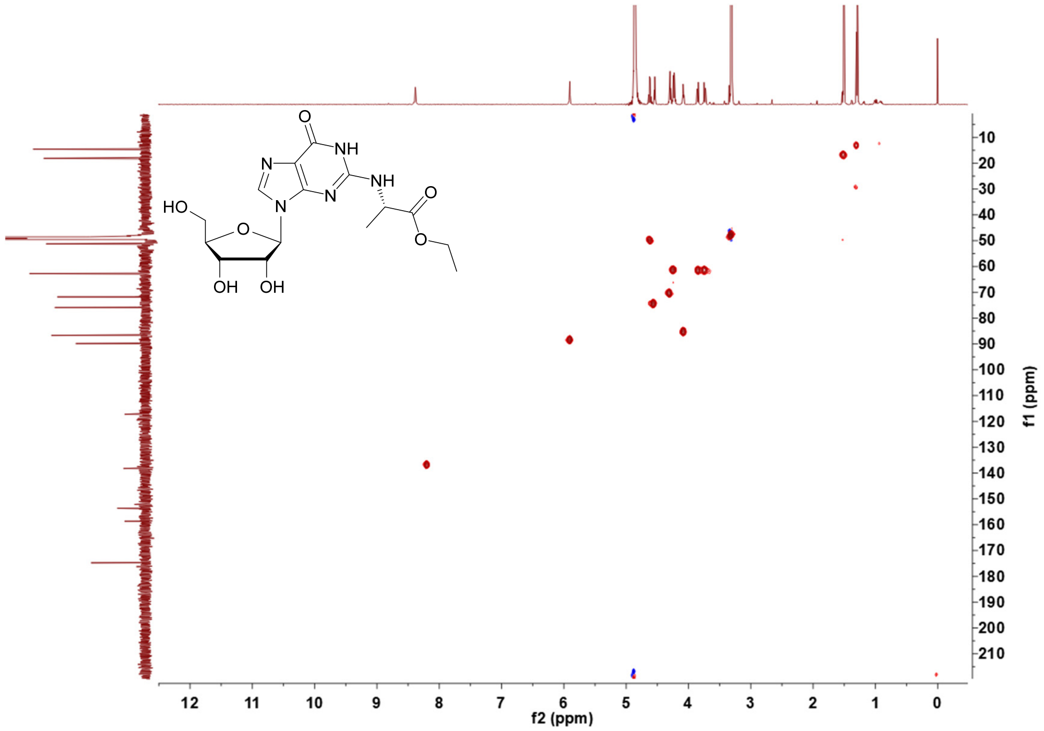

Figure S39. HSQC spectrum of **7** in methanol-d_4.

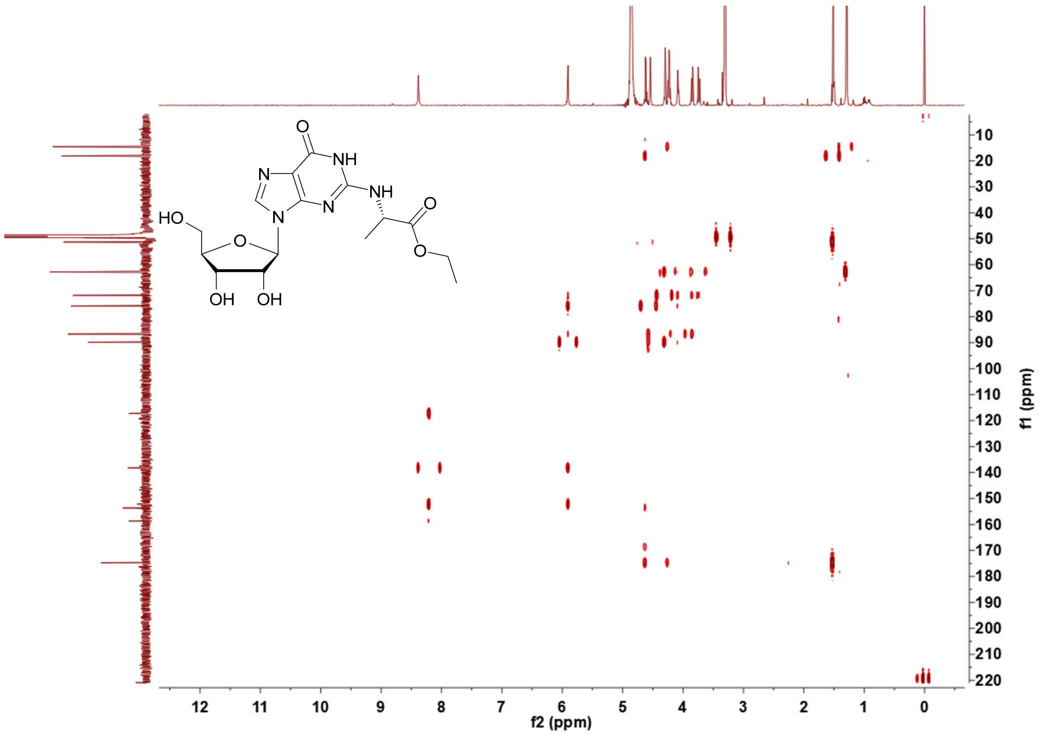

Figure S40. HMBC spectrum of **7** in methanol-d_4.

Acquisition Date	29/8/2020 2:38:55 PM	Result Table	XXC-145
Acquisition Method	N/A	Algorithm Used	AutoPeak
Project	N/A	Instrument Name	X500 QTOF

Mass Spretra

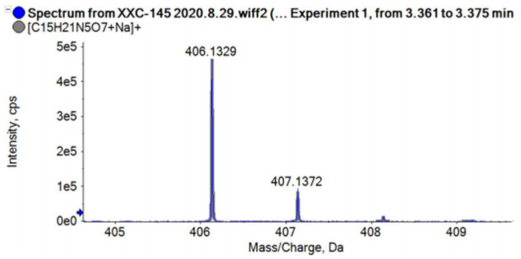

#	Analyte Peak Name	Formula	Precursor Mass	Found At Mass	Mass Error (ppm)
1	**XXC-145**	C15H21N5O7	406.1330	406.1329	-1.0

Figure S41. HRESIMS of **7**.

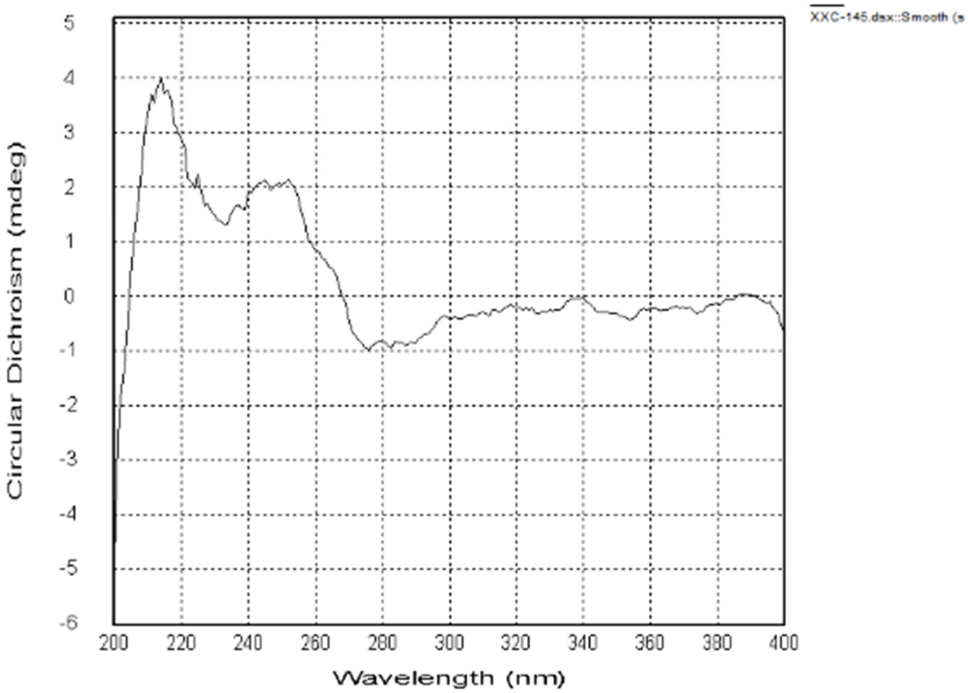

Figure S42. CD spectrum of **7** in methanol.

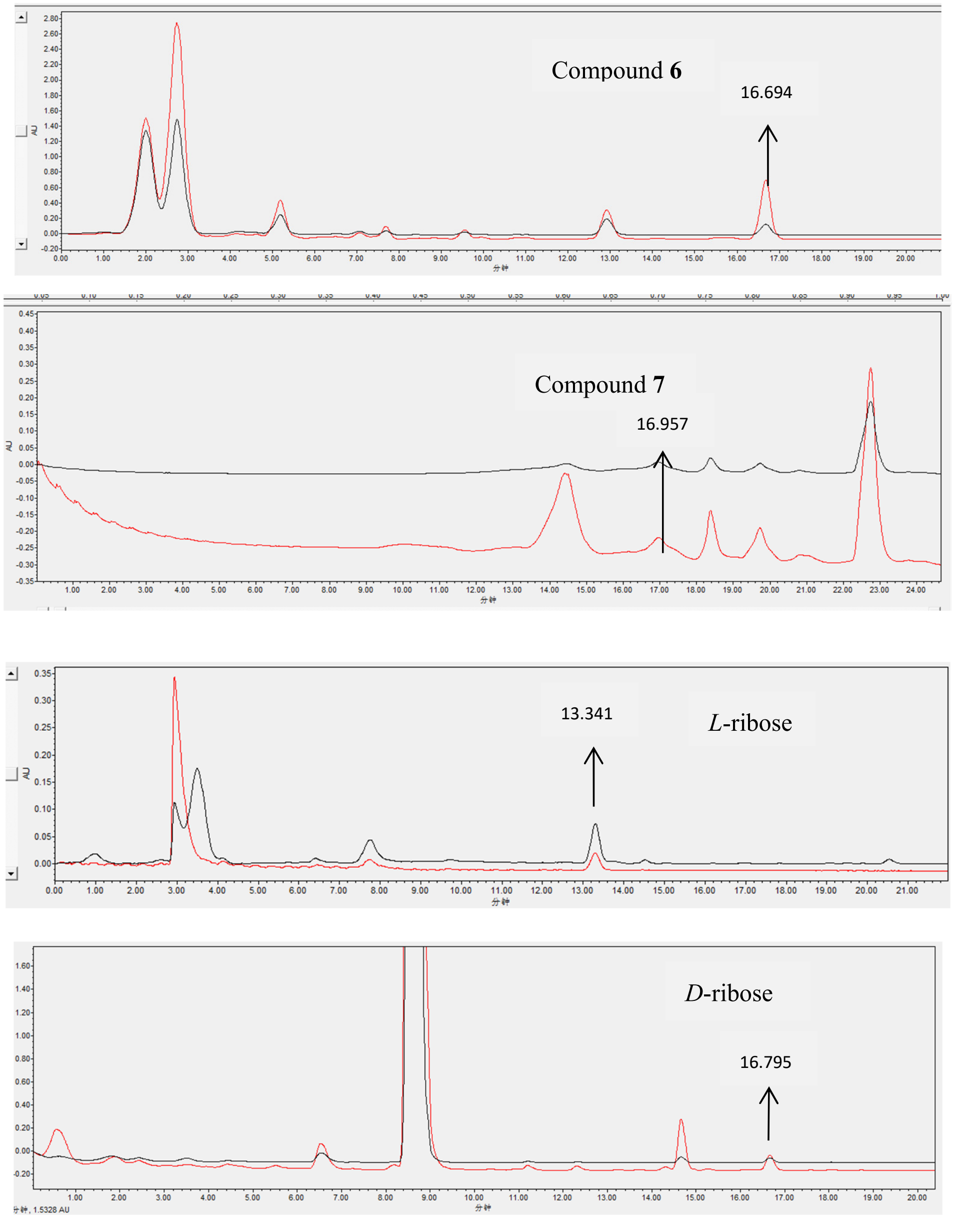

Figure S43. HPLC analysis of the derivatives of compounds **6**, **7**, *D*-ribose, and *L*-ribose (the red trace (210 nm),the black trace (254 nm))

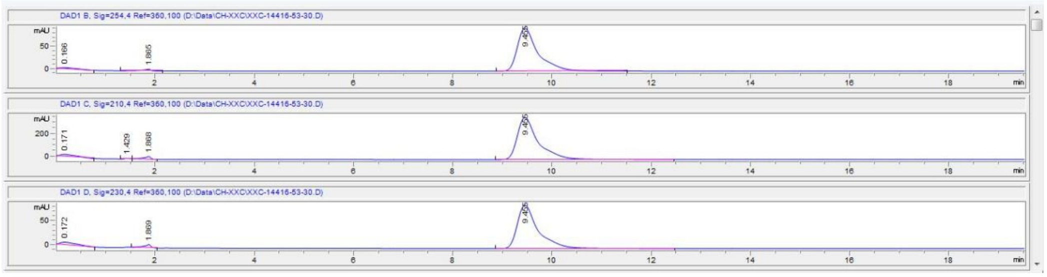

Figure S44. The chiral HPLC analysis of **6** by Daicel Chiralpak AD-H column.

(250 mm × 4.6 mm, i.d., 5 μm)

Analysis condition: n-hexane: ethanol 77:23 (flow rate: 1 mL/min).

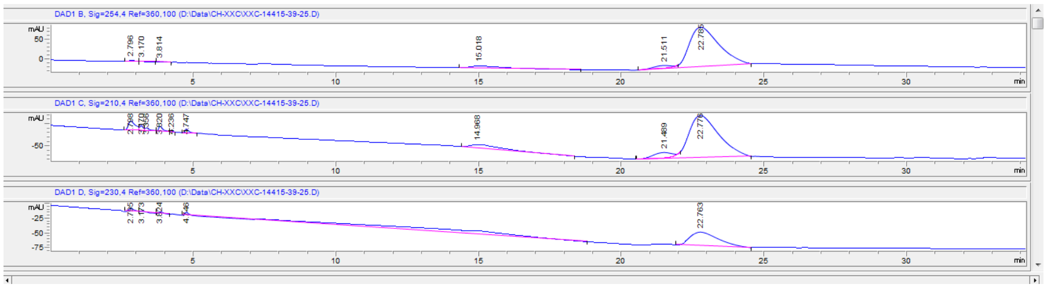

Figure S45. The chiral HPLC analysis of **6** by Daicel Chiralpak IC column.

(250 mm × 4.6 mm, i.d., 5 μm)

Analysis condition: n-hexane: ethanol 77:23 (flow rate: 1 mL/min).

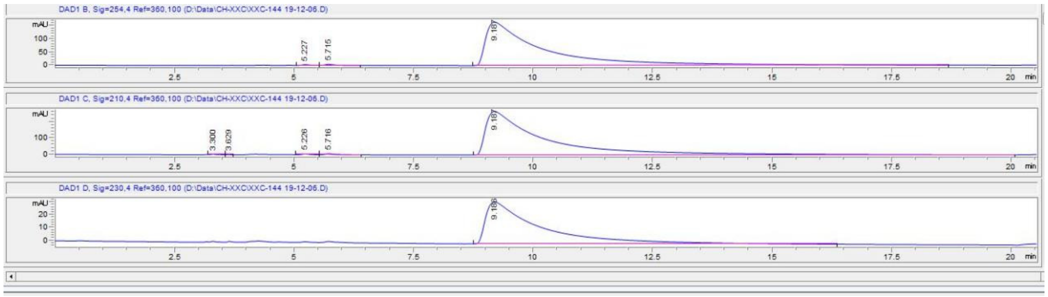

Figure S46. The chiral HPLC analysis of **6** by Daicel Chiralpak OD-H column.

(250 mm × 4.6 mm, i.d., 5 μm)

Analysis condition: n-hexane: ethanol 76:24 (flow rate: 1 mL/min).

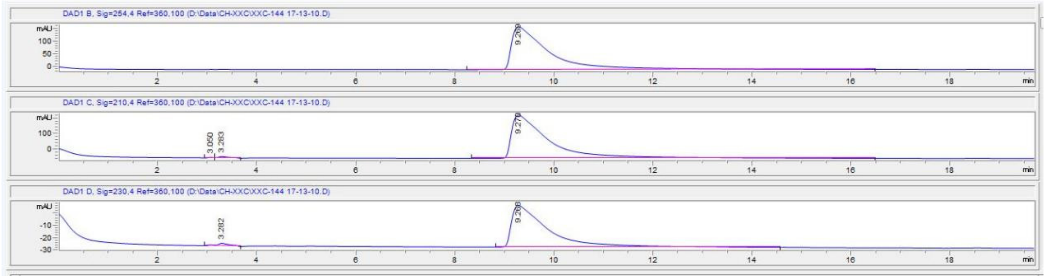

Figure S47. The chair HPLC analysis of **6** by Daicel Chiralpak Phenomenex column.

(OOG-4762-E0 LUX® i-Amylose-1, 250 mm × 4.6 mm, i.d., 5 μm)

Analysis condition: n-hexane: ethanol 77:23 (flow rate: 1 mL/min).

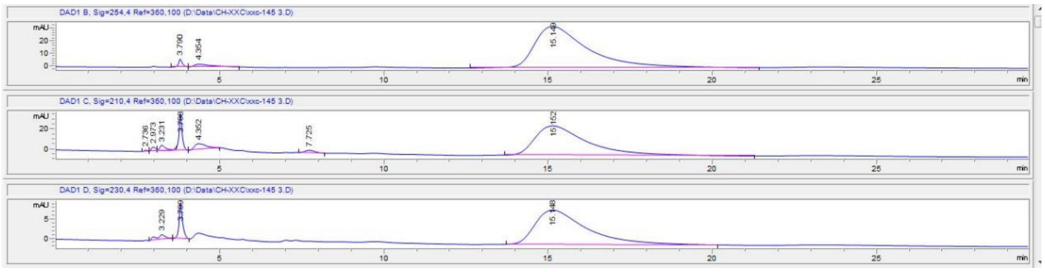

Figure S48. The chiral HPLC analysis of 7 by Daicel Chiralpak OD-H column.

(250 mm × 4.6 mm, i.d., 5 μm)

Analysis condition: n-hexane: ethanol 80:20 (flow rate: 1 mL/min).

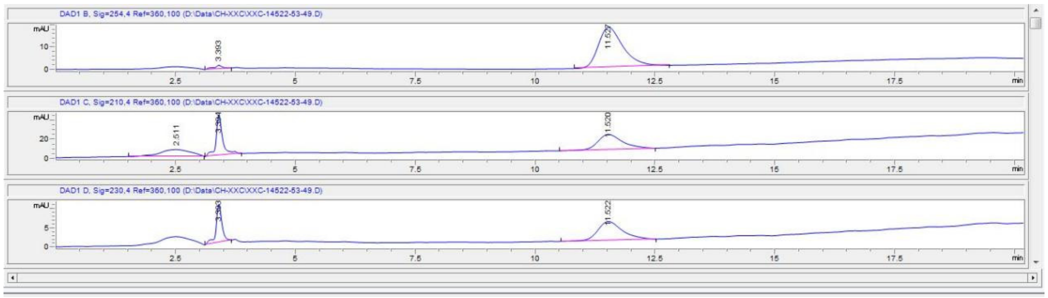

Figure S49. The chiral HPLC analysis of **7** by Daicel Chiralpak IC column.

(250 mm × 4.6 mm, i.d., 5 *μ*m)

Analysis condition: n-hexane: ethanol 57:43 (flow rate: 1 mL/min).

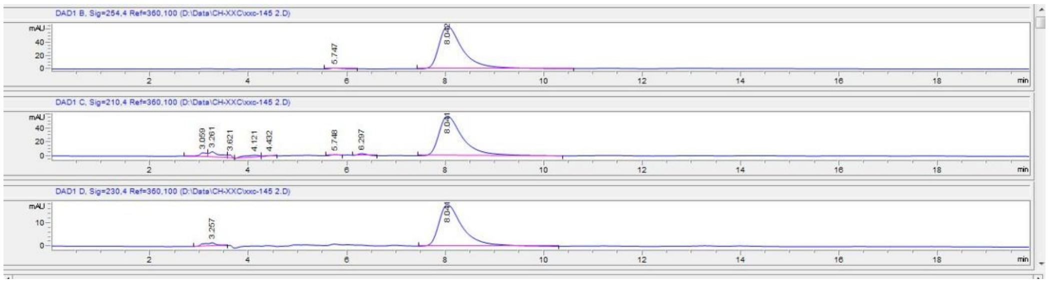

Figure S50. The chiral HPLC analysis of **7** by Daicel Chiralpak AD-H column.

(250 mm × 4.6 mm, i.d., 5 *μ*m)

Analysis condition: n-hexane: ethanol 75:25 (flow rate: 1 mL/min).

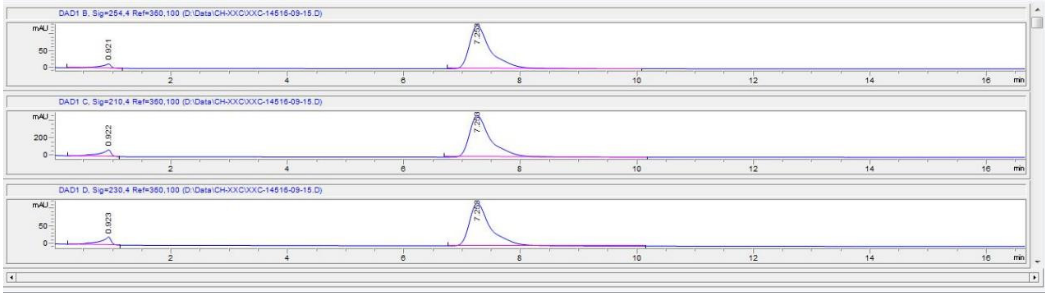

Figure S51. The chair HPLC analysis of **7** by Daicel Chiralpak Phenomenex column.

(OOG-4762-E0 LUX® i-Amylose-1, 250 mm × 4.6 mm, i.d., 5 μm)

Analysis condition: n-hexane: ethanol 75:25 (flow rate: 1 mL/min).

Compounds	50 μM	25 μM	12.5 μM	Results
1a	++++	++++	++++	×
1b	++++	++++	++++	×
2a	++++	++++	++++	×
2b	++++	++++	++++	×
3a	++++	++++	++++	×
3b	++++	++++	++++	×
4a	++++	++++	++++	×
4b	++++	++++	++++	×
5a	++++	++++	++++	×
5b	++++	++++	++++	×

6	++++	++++	++++	×
7	++++	++++	++++	×

Figure S52. Anti-HSV-1/F activity screening results of compounds **1−7**. (*The recording method of CPE is: no cytopathy "-"; 1%~25% of cells with pathological changes are “+”; 26%~50% cytopathies are “++”; 51%~75% cytopathies are “+++”; 76%~100% of cell lesions are “++++”. “√” indicates antiviral activity, “×” indicates no antiviral activity).

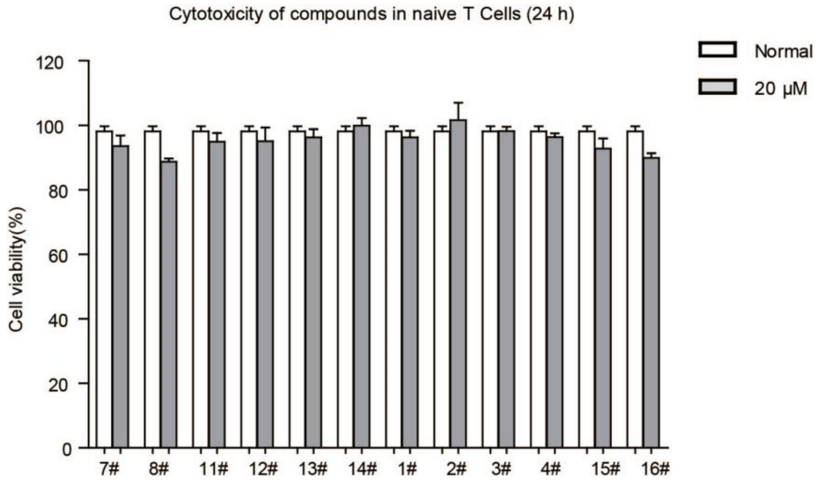

Figure S53. Cytotoxicity of compounds **1−7** in naive T Cells. (No.7#, 8#, 11#, 12#, 13#, 14#, 1#, 2#, 3#, 4#, 15#, 16# in the figure are compounds **1a**, **1b**, **2a**, **2b**, **3a**, **3b**, **4a**, **4b**, **5a**, **5b**, **6**, **7** respectively).

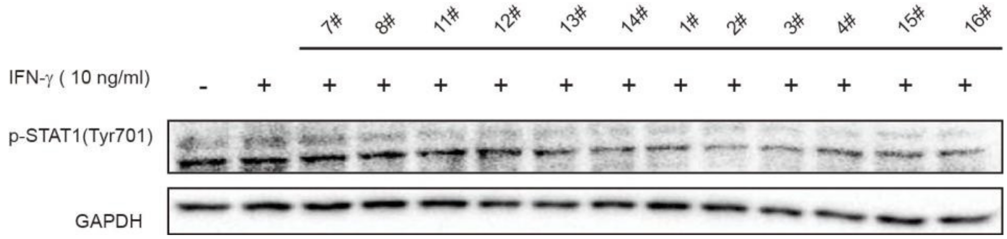

Figure S54. Effect of compounds **1−7** on IFN- γ signaling in mouse T cells in vitro. (No.7#, 8#, 11#, 12#, 13#, 14#, 1#, 2#, 3#, 4#, 15#, 16# in the figure are compounds **1a**, **1b**, **2a**, **2b**, **3a**, **3b**, **4a**, **4b**, **5a**, **5b**, **6**, **7** respectively).

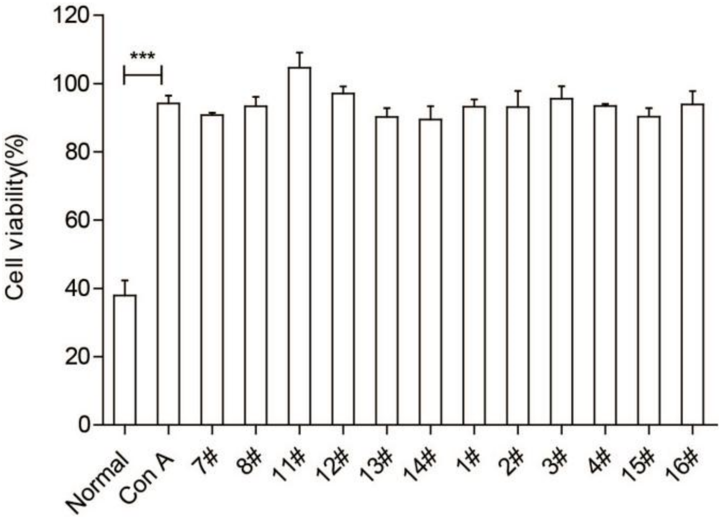

Figure S55. Effect of compounds **1−7** on T-cell proliferation and activation activated by Con A. (No.7#, 8#, 11#, 12#, 13#, 14#, 1#, 2#, 3#, 4#, 15#, 16# in the figure are compounds **1a**, **1b**, **2a**, **2b**, **3a**, **3b**, **4a**, **4b**, **5a**, **5b**, **6**, **7** respectively).

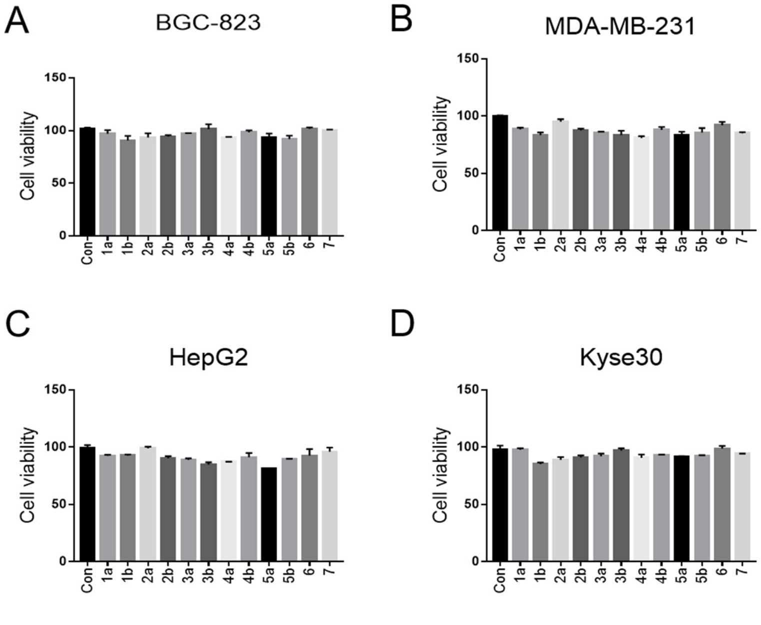

Figure S56. Cytotoxic effects of the above compounds on human cancer cells (A, B, C, D).
